# Supplementary figures and images for: Fine scale mapping of genomic introgressions within the Drosophila yakuba clade
Source: PLoS Genet. 2017 Sep 5;13(9):e1006971. doi: 10.1371/journal.pgen.1006971 (PMC5600410; doi:10.1371/journal.pgen.1006971)

A)

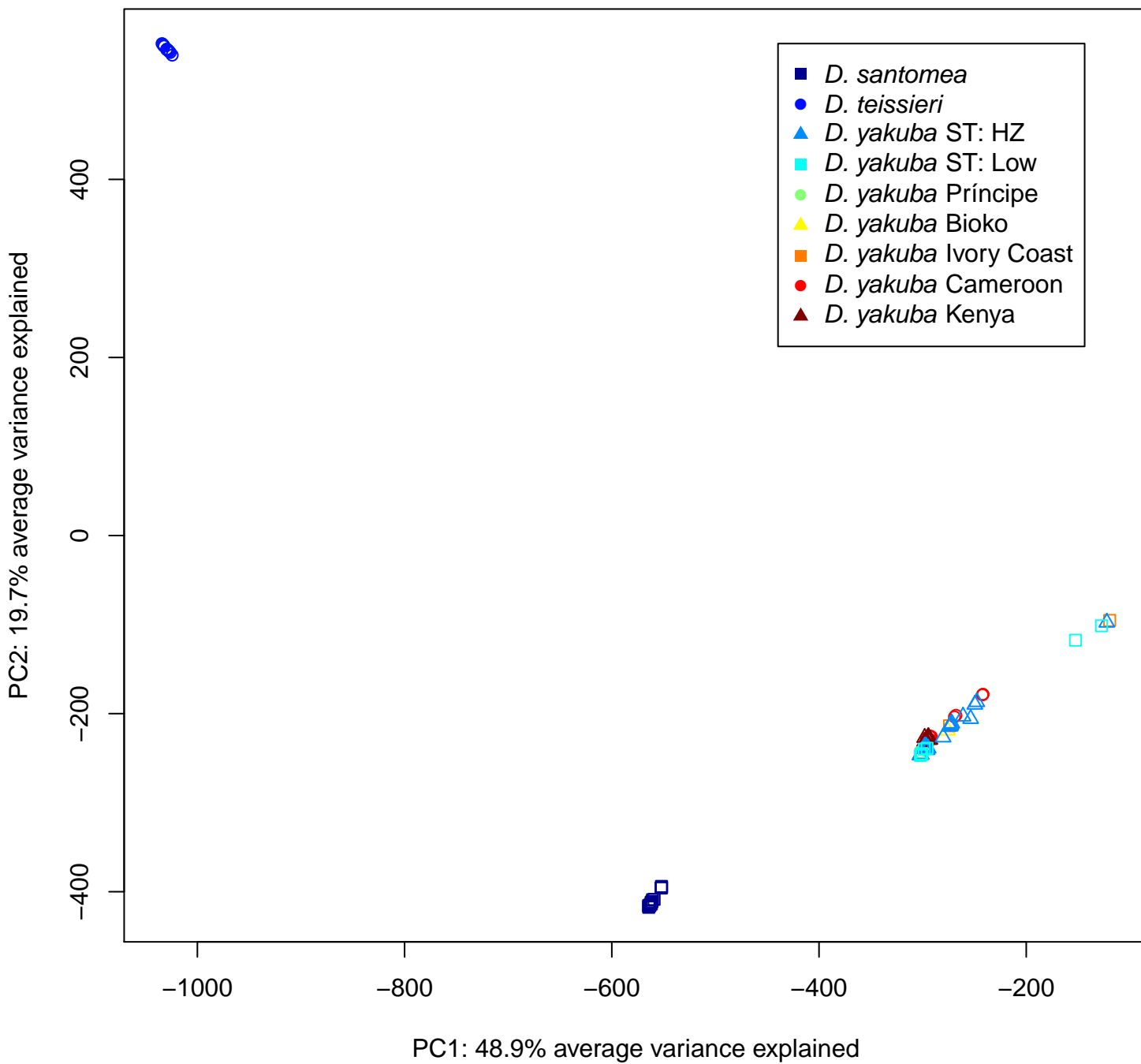

B)

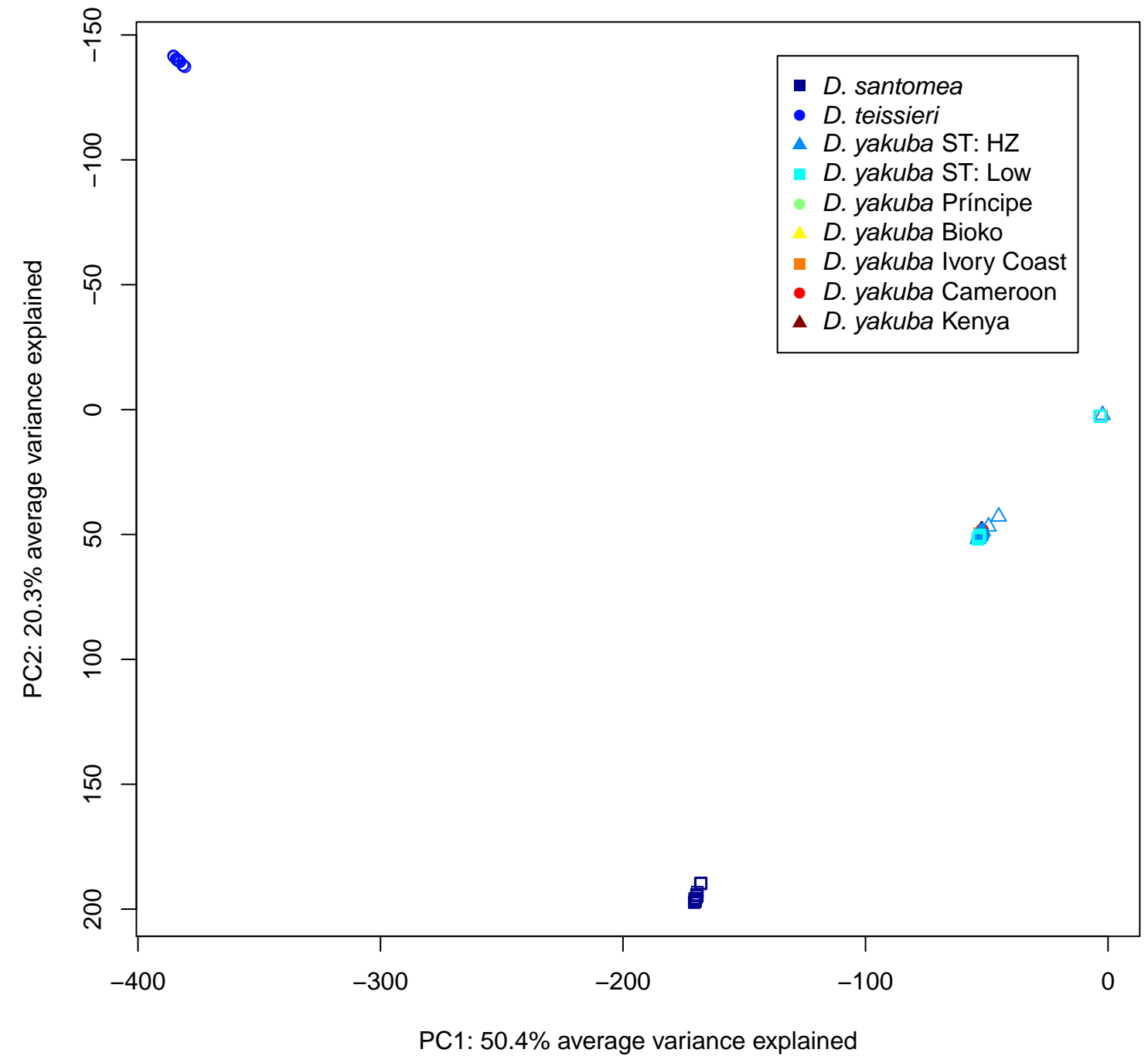

Supplement: S1 Fig — Principle component results for PC1 and PC2 for the D. yakuba clade. A) Autosomes. B) X chromosome. (PDF) [file pgen.1006971.s001.pdf]

A)

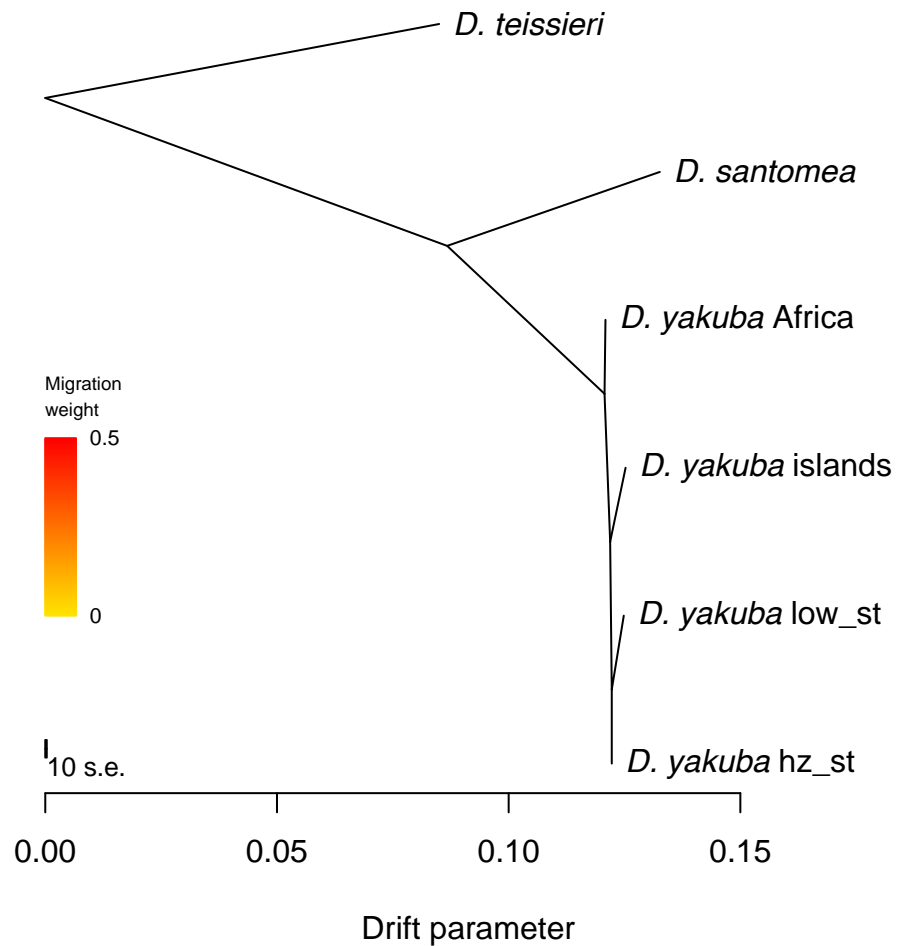

B)

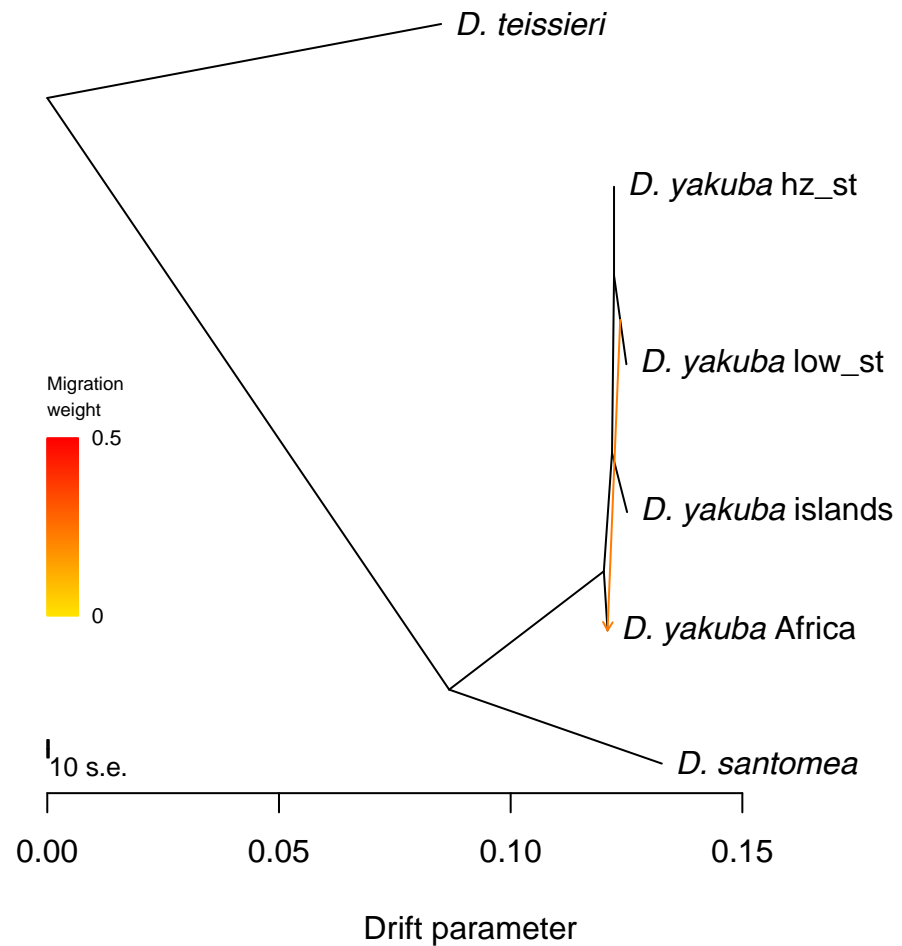

Supplement: S2 Fig — X chromosome Treemix trees with 0 to 3 migration edges (the most likely value of m = 4, Fig 1A). Drosophila yakuba was split into four populations: “africa” (Cameroon, Kenya, Ivory Coast), “islands” (Príncipe and Bioko), “low_st” (lowlands of São Tomé), and “hz_st” (hybrid zone on São Tomé). The P value was calculated for a tree with m migration edges by taking a log-likelihood ratio test using the likelihoods for the threes with m and m-1 migration edges. A) m = 0. B) m = 1. C) m = 2. D) m = 3. (PDF) [file pgen.1006971.s002.pdf]

A)

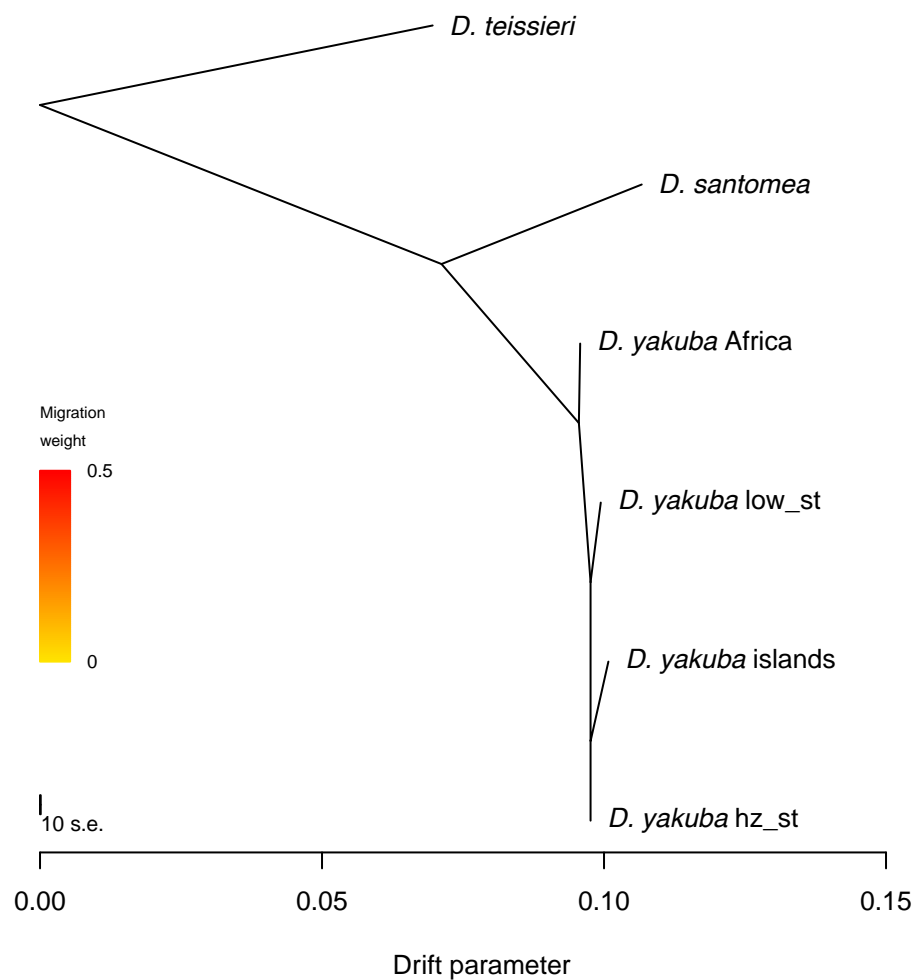

B)

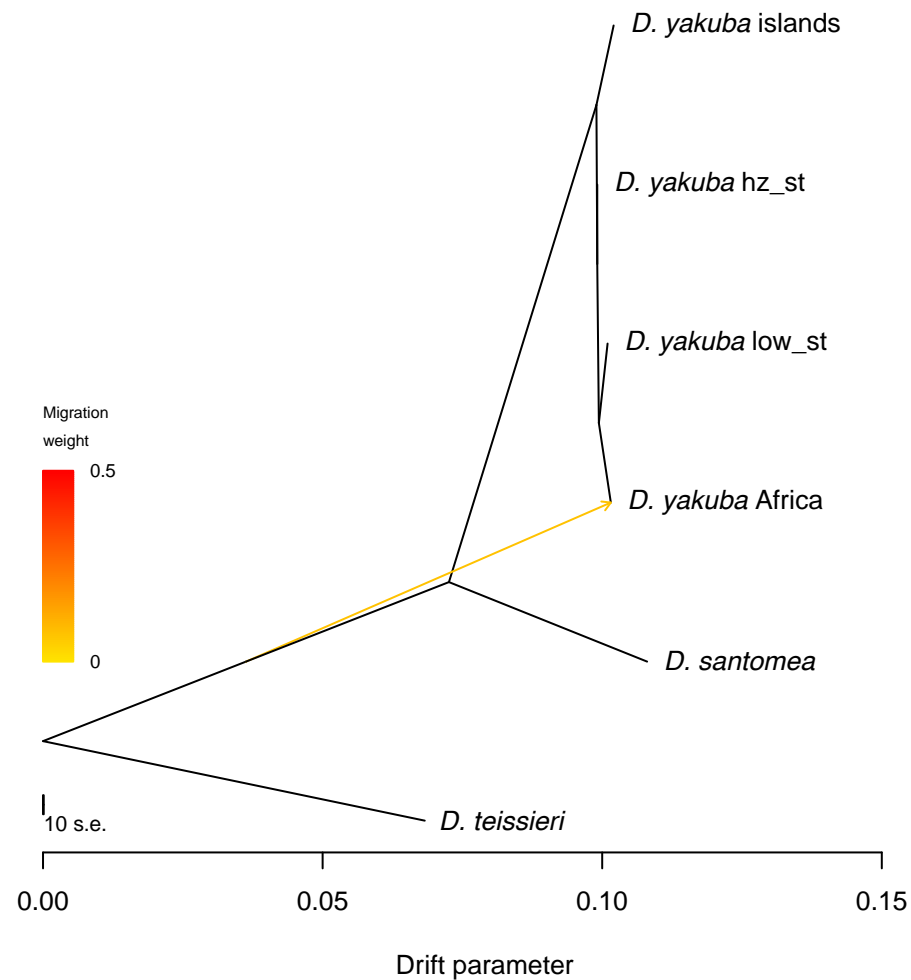

C)

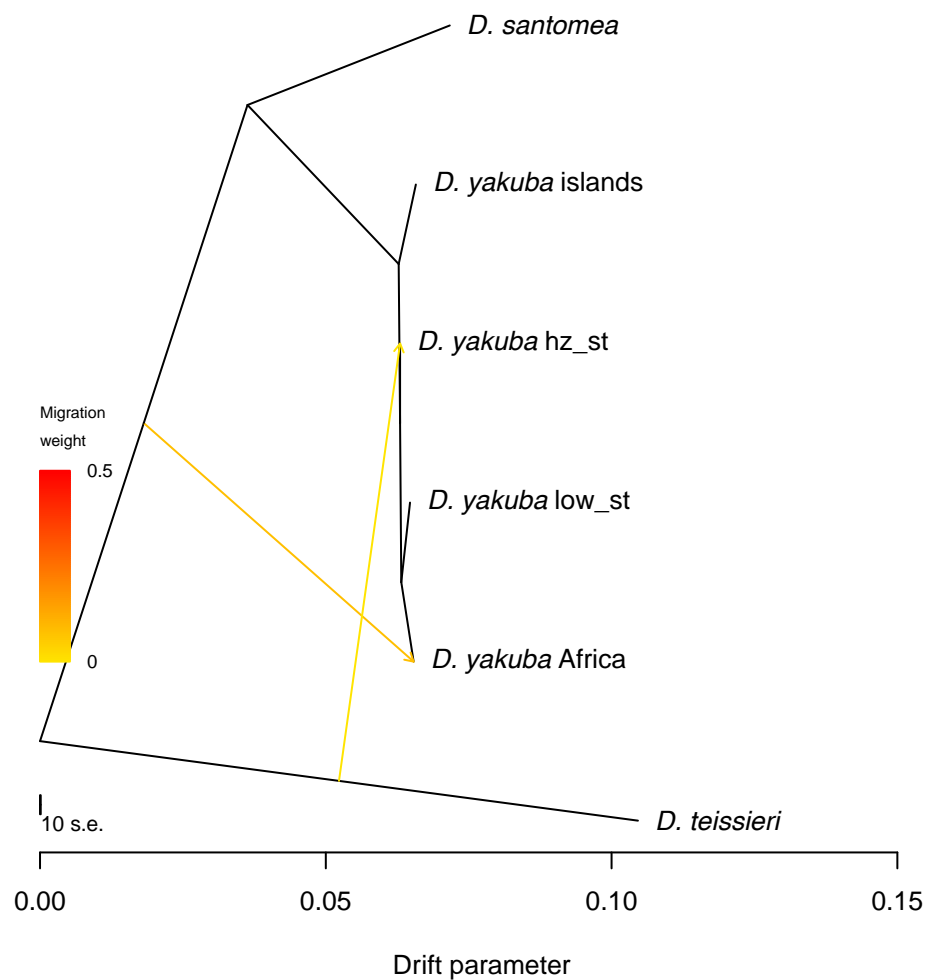

D)

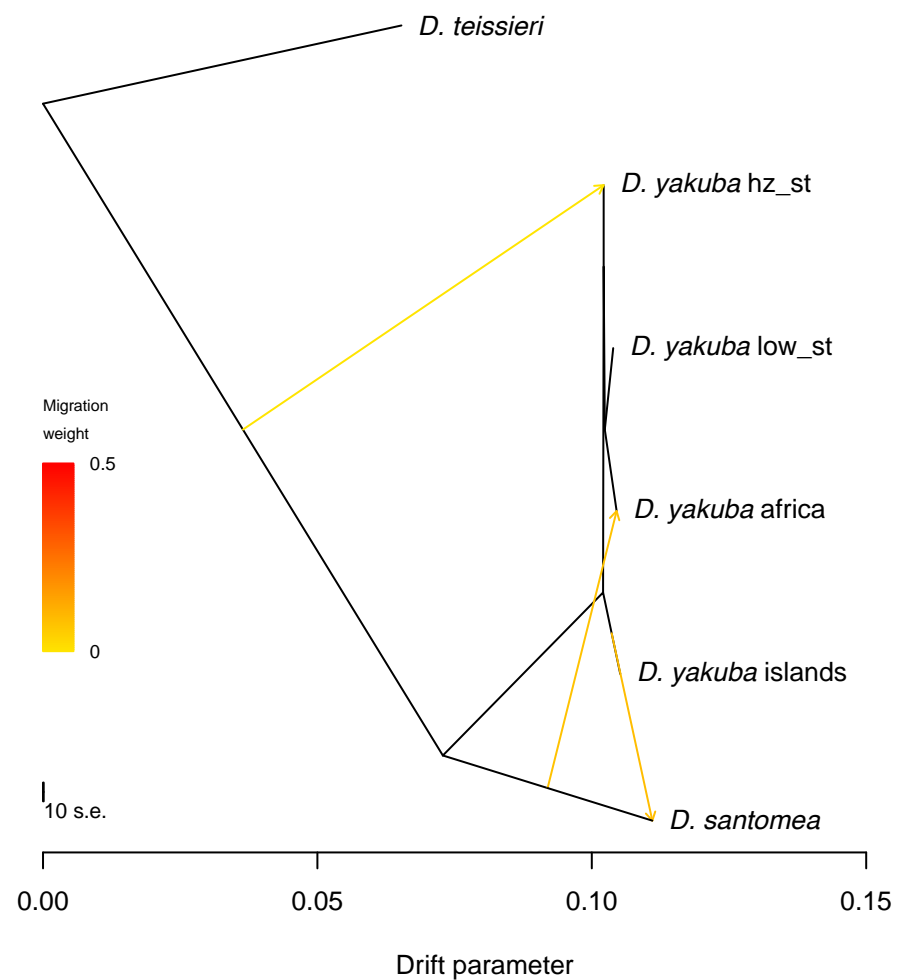

Supplement: S3 Fig — Autosomal Treemix trees with 0 to 3 migration edges (the most likely value of m = 4, Fig 1B). Drosophila yakuba was split into four populations: “africa” (Cameroon, Kenya, Ivory Coast), “islands” (Príncipe and Bioko), “low_st” (lowlands of São Tomé), and “hz_st” (hybrid zone on São Tomé). The P value was calculated for a tree with m migration edges by taking a log-likelihood ratio test using the likelihoods for the threes with m and m-1 migration edges. A) m = 0. B) m = 1. C) m = 2. D) m = 3. (PDF) [file pgen.1006971.s003.pdf]

A)

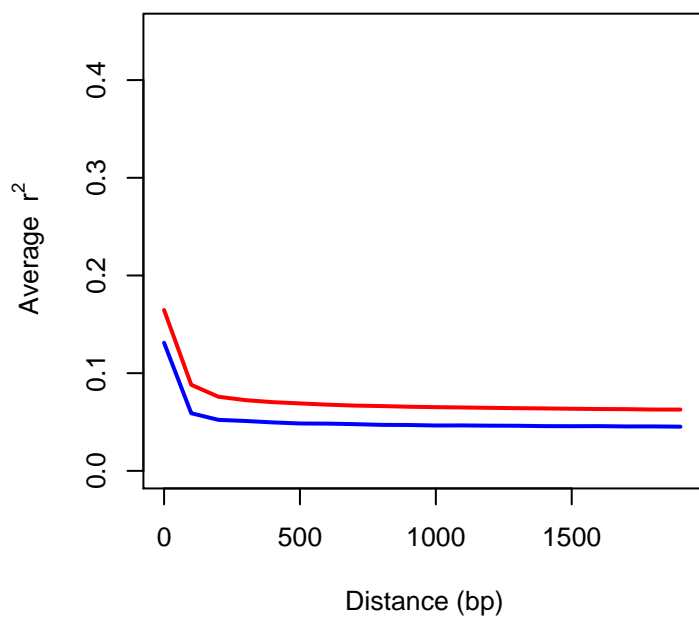

B)

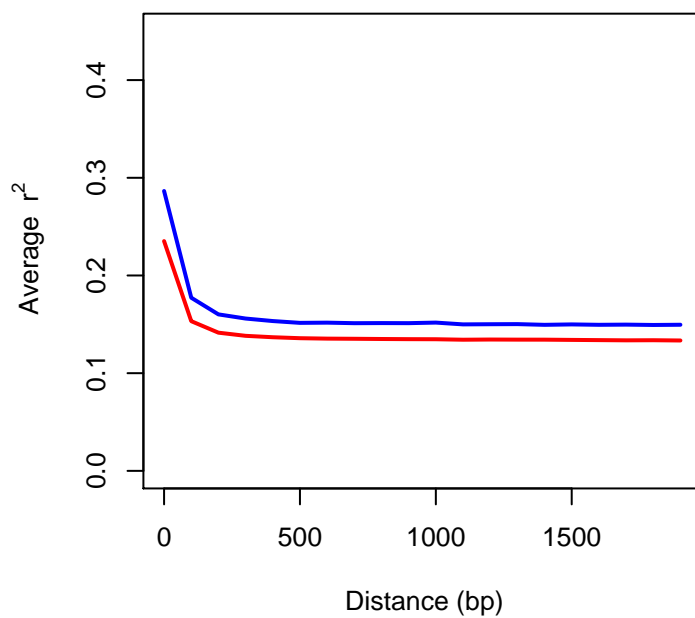

C)

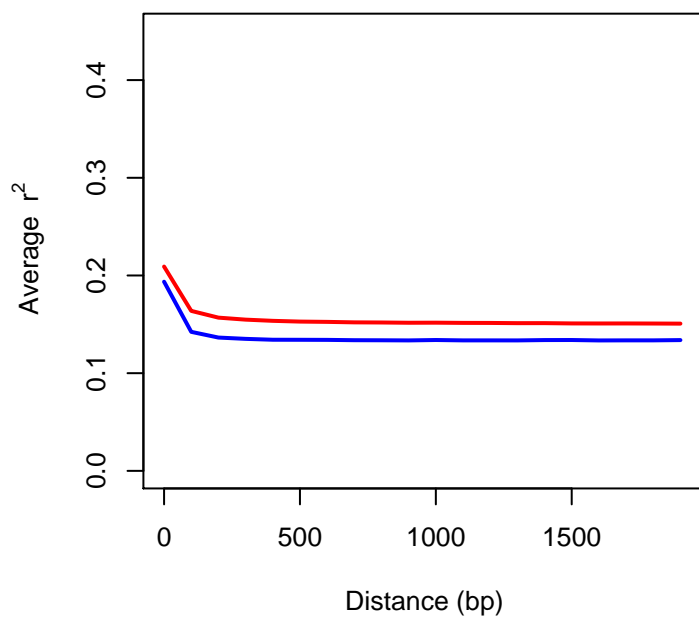

Autosomes  
X

Supplement: S4 Fig — Average LD as measured by r2 between pairs of SNPs with distances binned every 100bp. r2 was averaged separately for the autosomes (red) and X chromosome (blue). A) D. yakuba. B) D. santomea. C) D. teissieri. (PDF) [file pgen.1006971.s004.pdf]

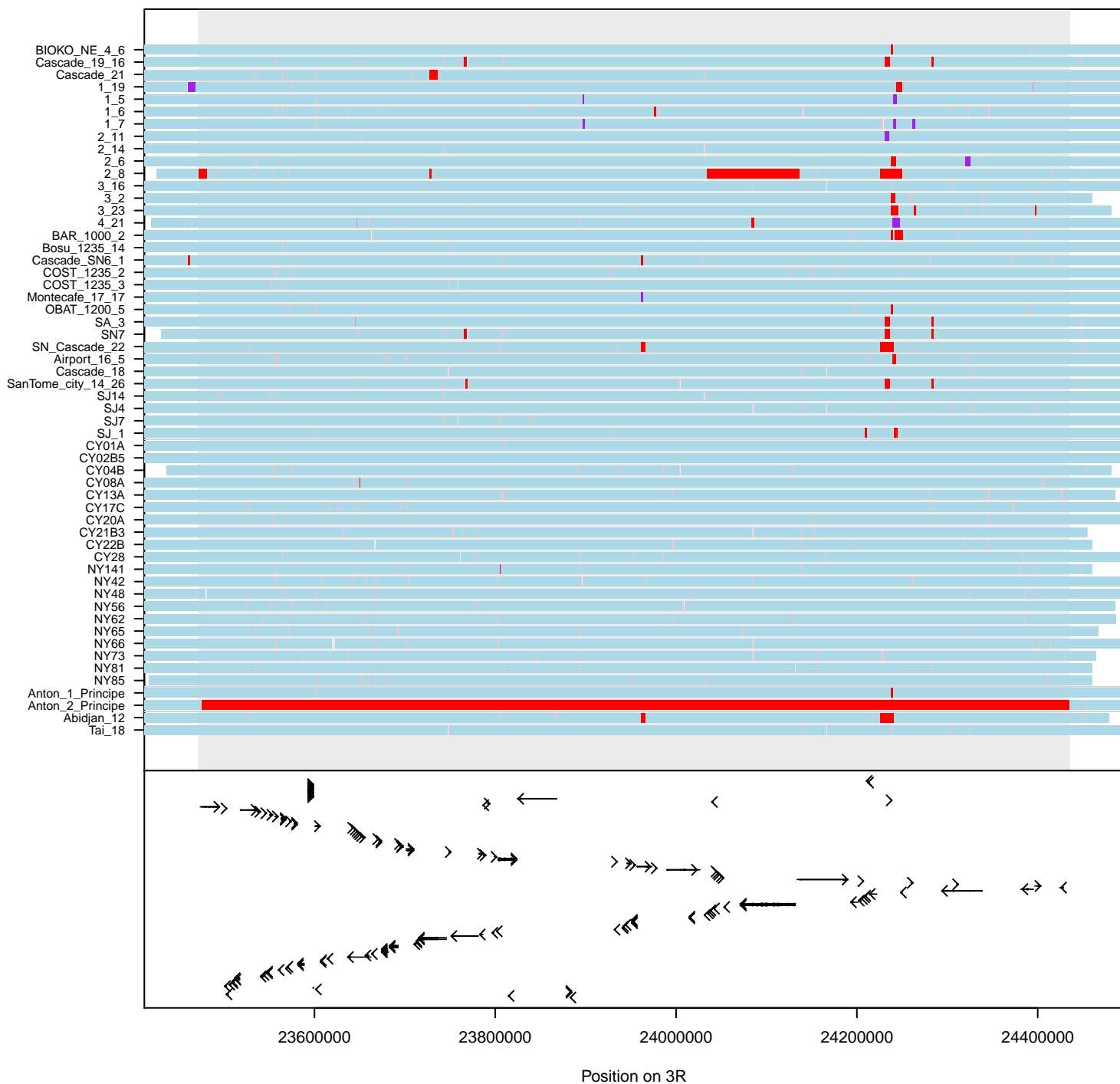

Supplement: S5 Fig — 959kb introgression from D. santomea into the D. yakuba line Anton_2_Principe collected from the island of Príncipe that is significantly larger than the second biggest introgression (120kb). Red denotes homozygous D. santomea tracts, light blue tracts are homozygous D. yakuba, and purple tracts are heterozygous (inferred using Int-HMM). The lower panel shows genes in the genomic region on 3R with the arrows denoting the direction of transcription. (PDF) [file pgen.1006971.s005.pdf]

A) *san*-into-*yak*  
P = 0

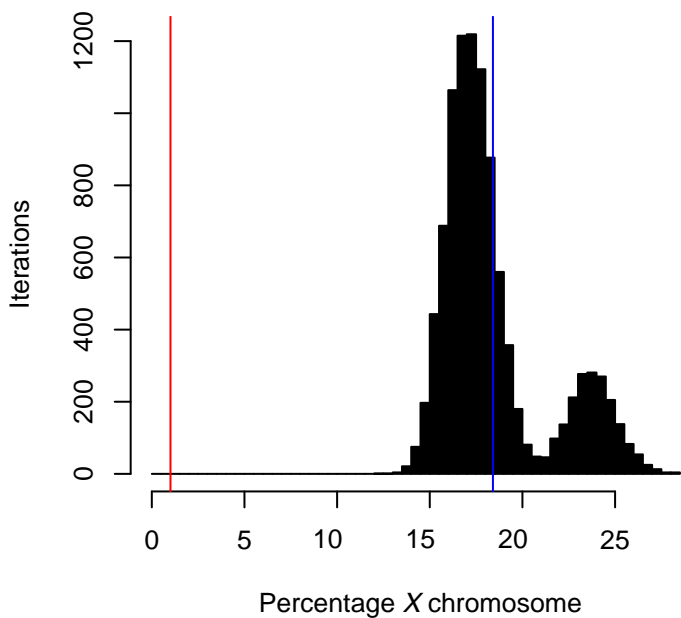

B) *yak*-into-*san*  
P = 0

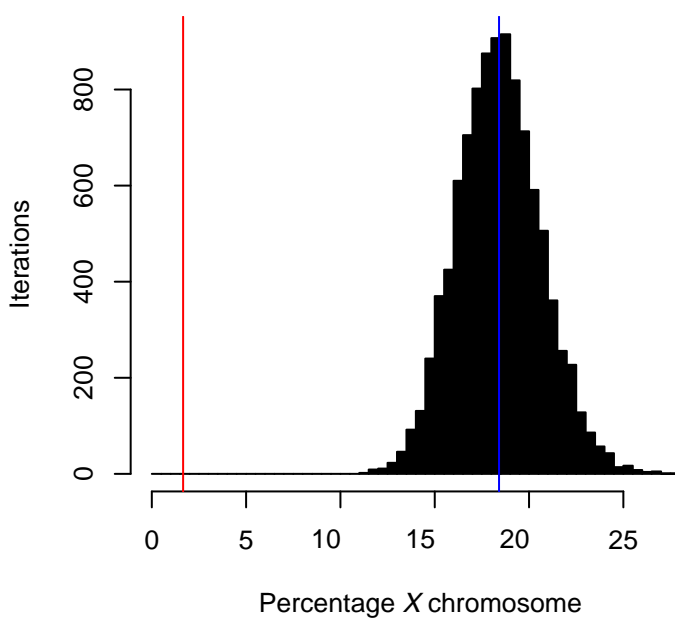

C) *tei*-into-*yak*  
P = 0

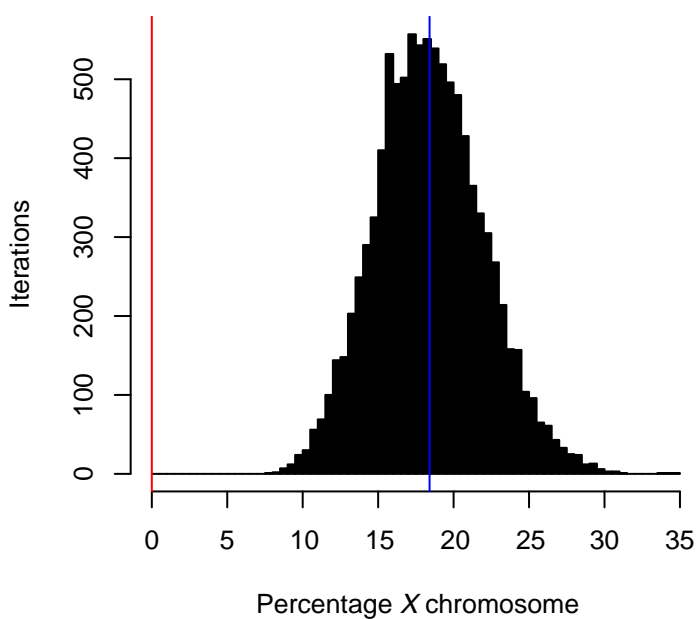

D) *yak*-into-*tei*  
P = 0.0221

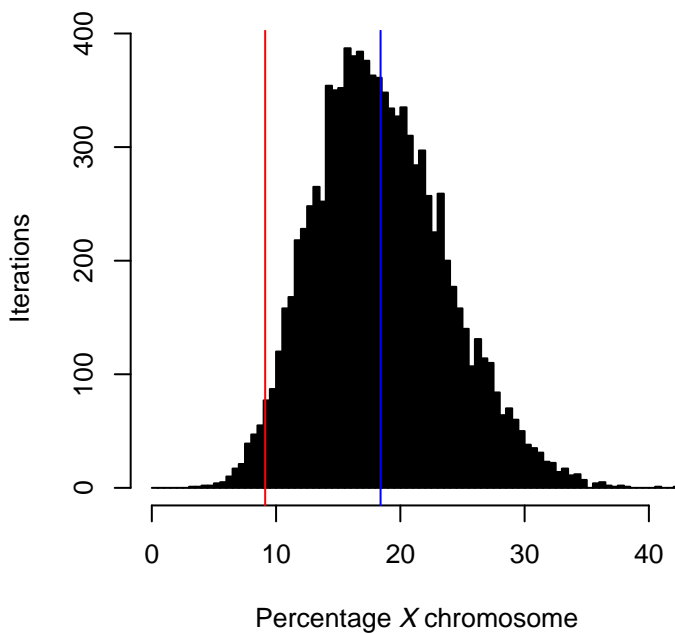

Supplement: S6 Fig — Percentage of introgressed sequence on the X chromosome for 10,000 iterations of resampling without replacement in a neutral scenario where introgressions are uniformly distributed across the genome. P values were obtained by dividing the number of resampled proportions that were lower than the observed value by 10,000. The red line indicates the observed percentage of introgressed sequence on the X chromosome, and the blue line is the average percentage from the 10,000 resampling iterations. A) san-into-yak. B) yak-into-san. C) tei-into-yak. D) yak-into-tei. (PDF) [file pgen.1006971.s006.pdf]

A)

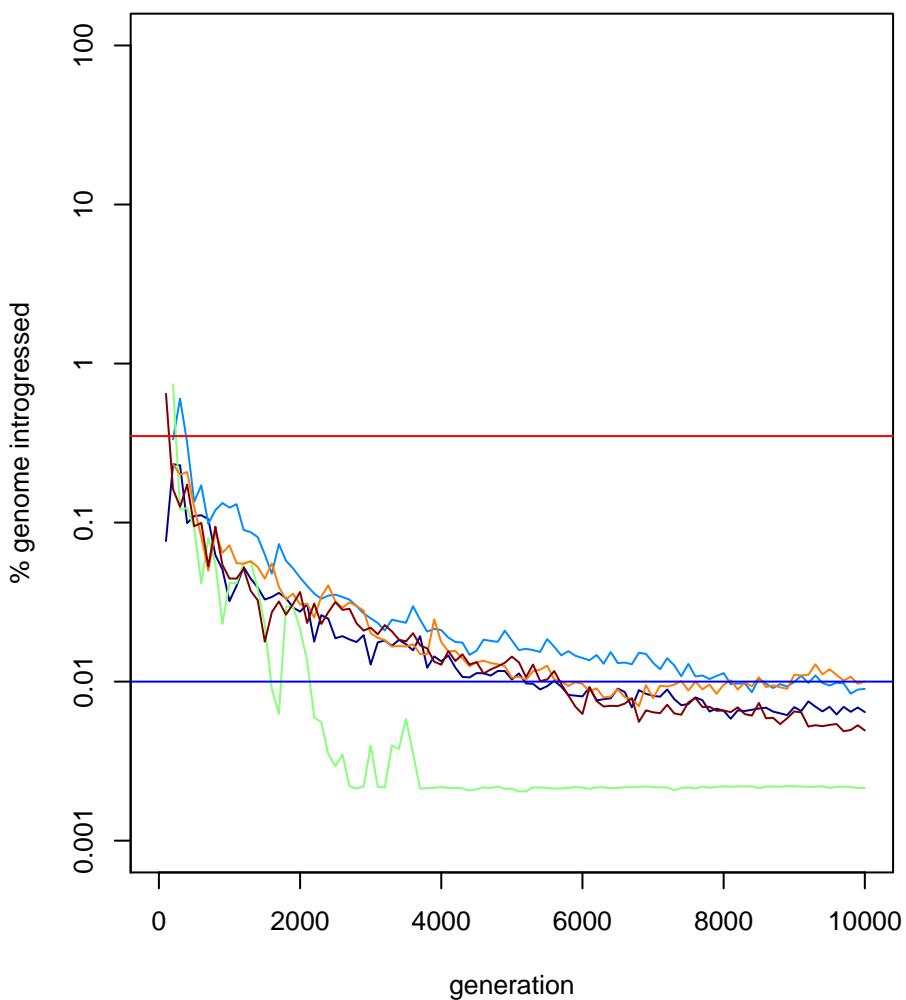

B)

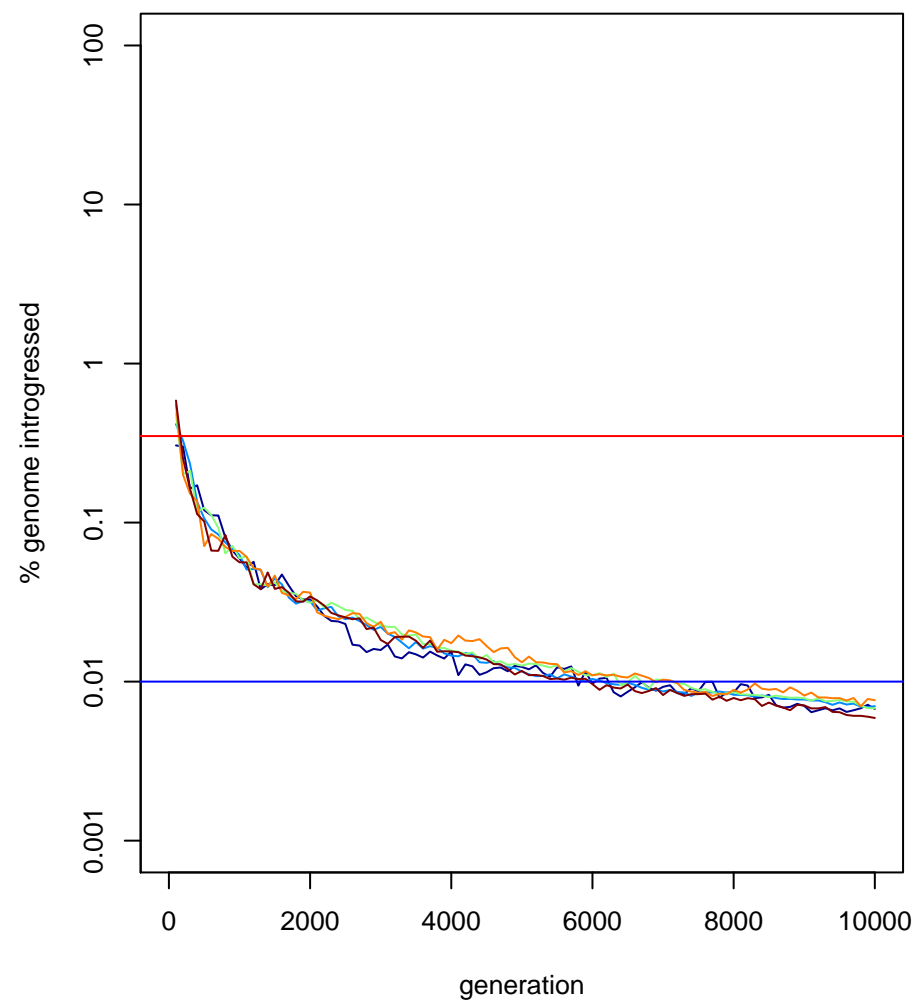

C)

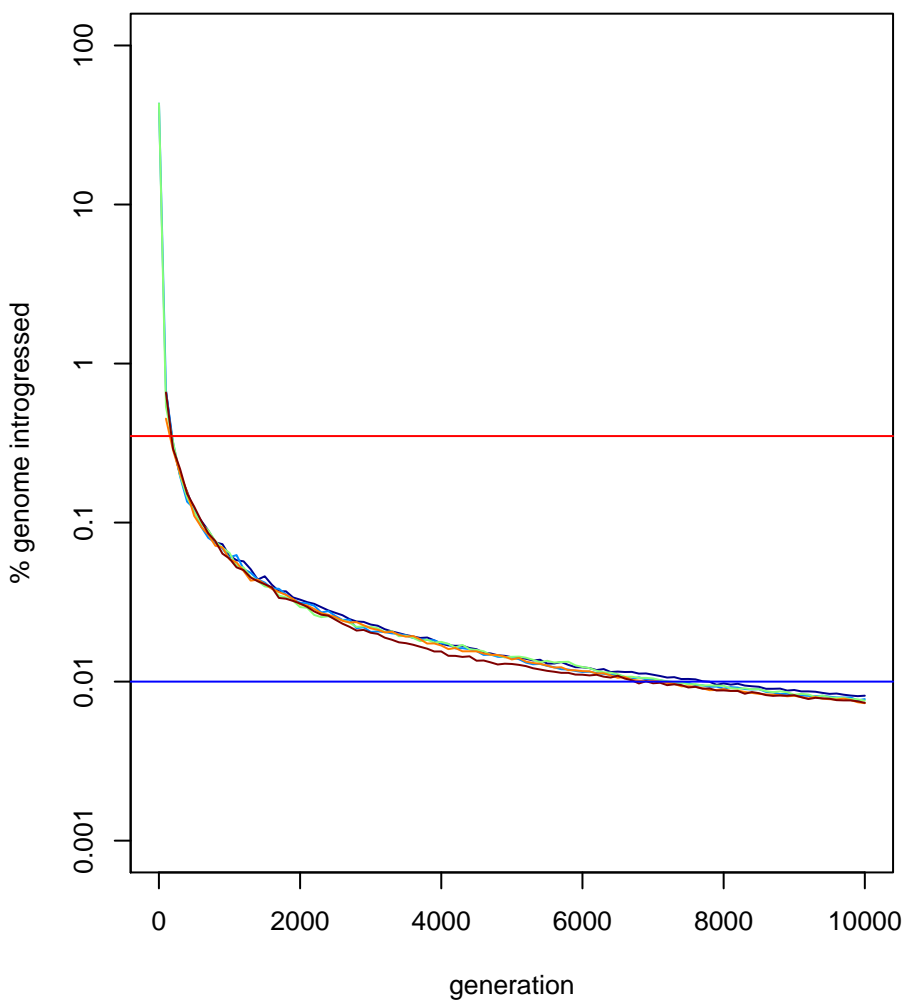

D)

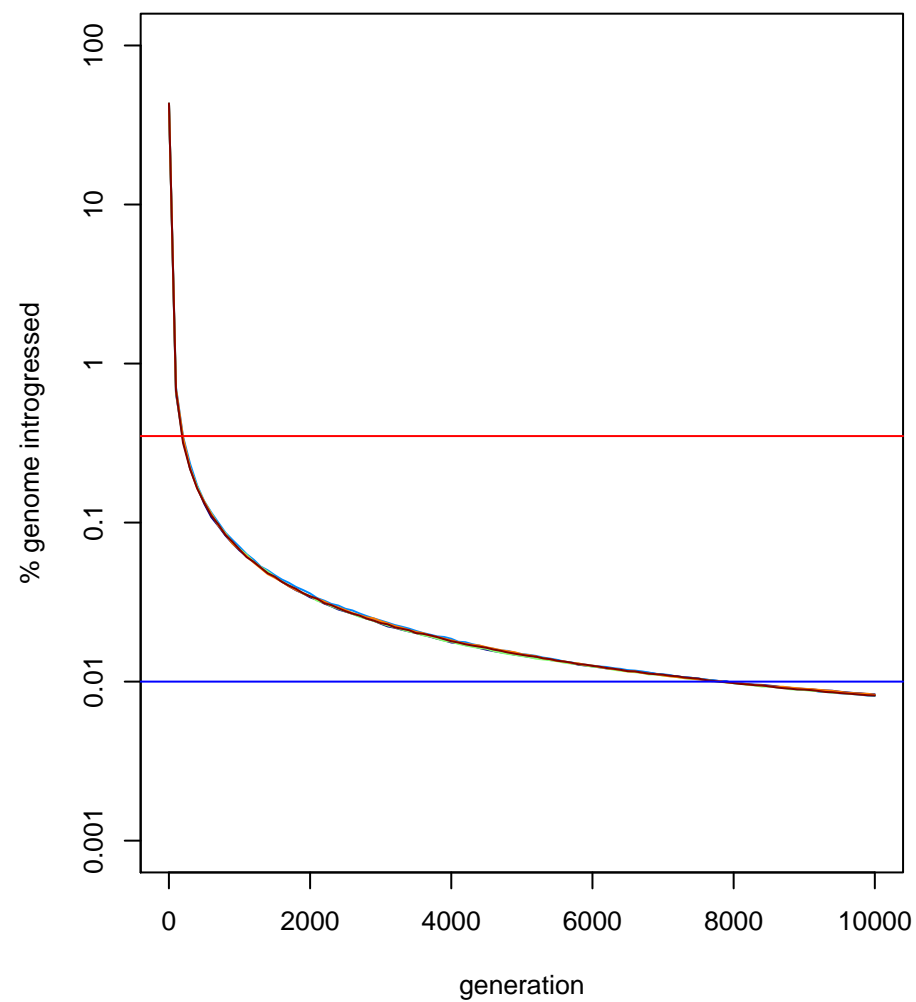

Supplement: S7 Fig — Results for five independent SELAM runs with a population size of 10,000 following a single generation of admixture. The horizontal red line represents the observed value for introgression between D. yakuba and D. santomea (0.35%), and the horizontal blue line denotes the observed value for introgression between D. yakuba and D. teissieri (0.01%). A) m = 0.0001. B) m = 0.001. C) m = 0.01. D) m = 0.1. (PDF) [file pgen.1006971.s007.pdf]

A)

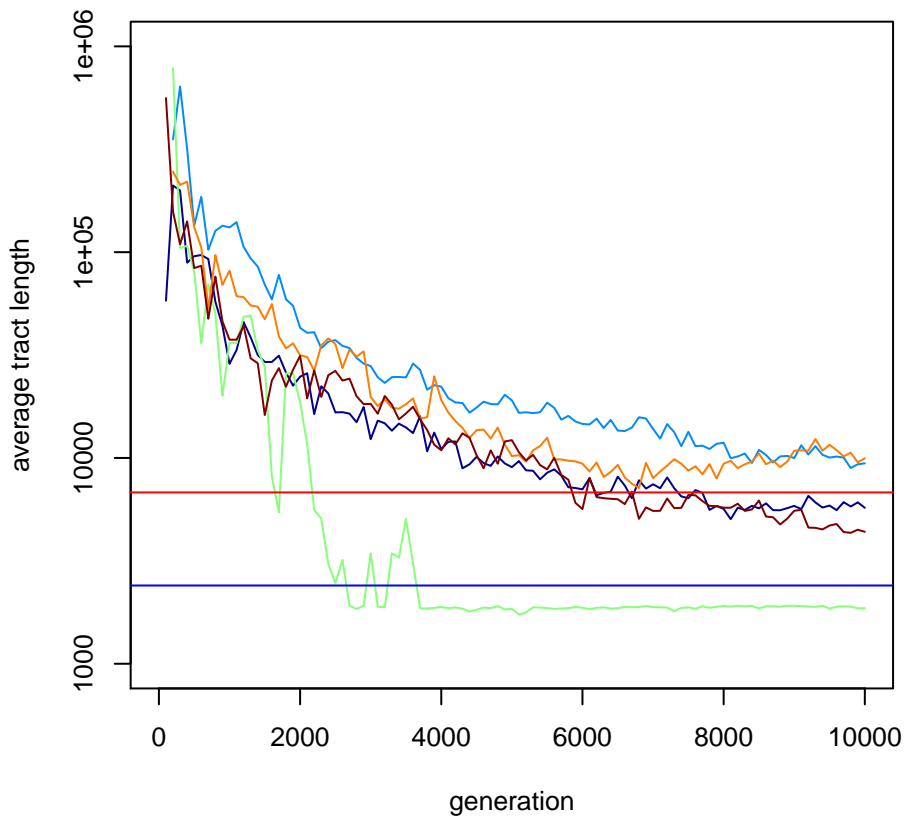

B)

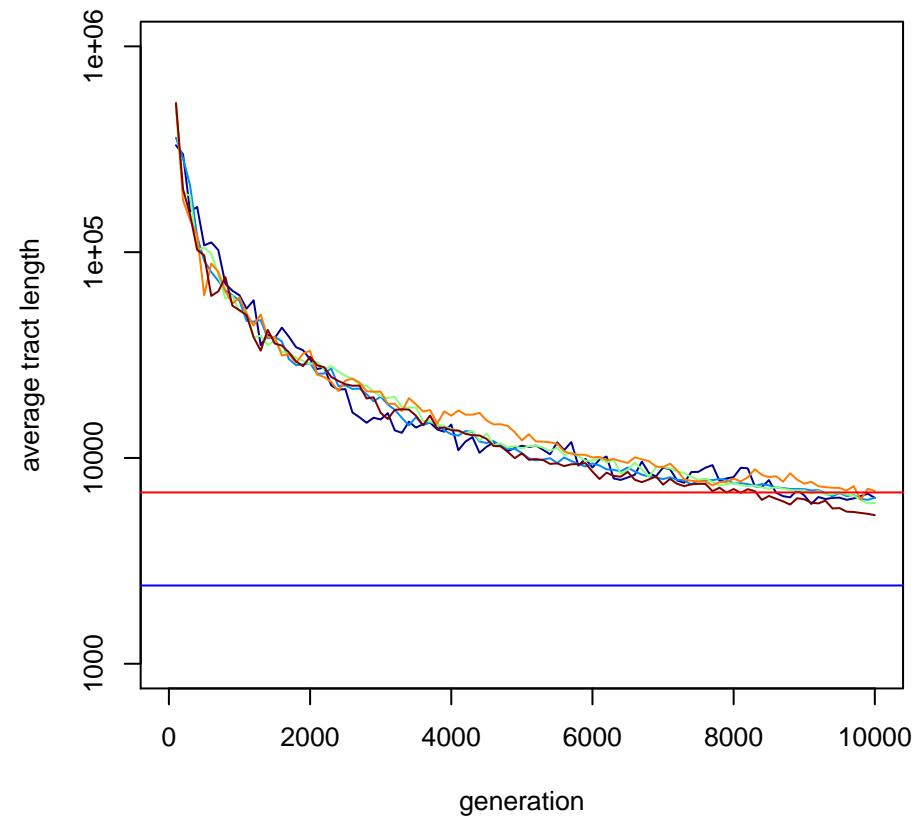

C)

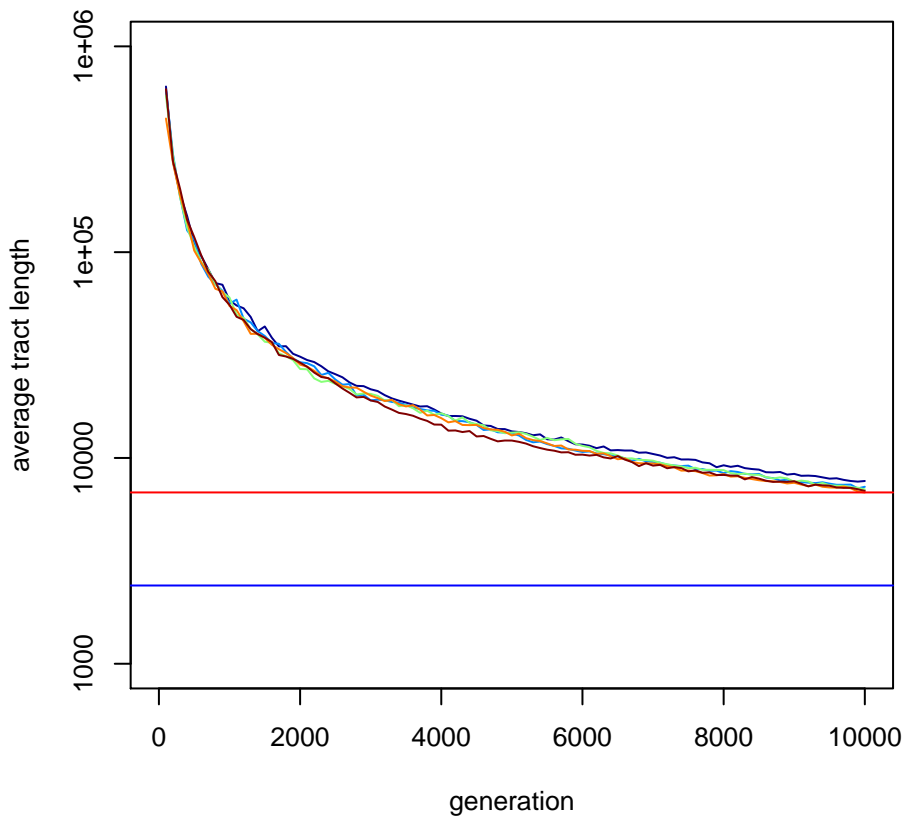

D)

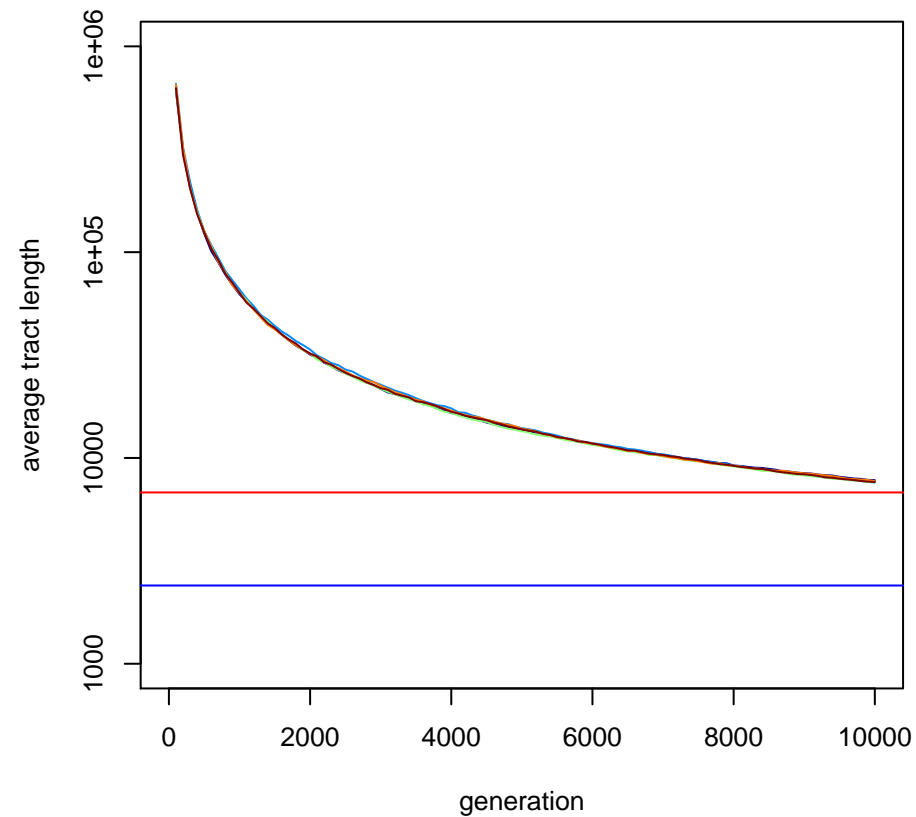

Supplement: S8 Fig — Results for five independent SELAM runs with a population size of 10,000 following a single generation of admixture. The horizontal red line represents the observed value for introgression between D. yakuba and D. santomea (6.8kb), and the horizontal blue line denotes the observed value for introgression between D. yakuba and D. teissieri (2.4kb). A) m = 0.0001. B) m = 0.001. C) m = 0.01. D) m = 0.1. (PDF) [file pgen.1006971.s008.pdf]

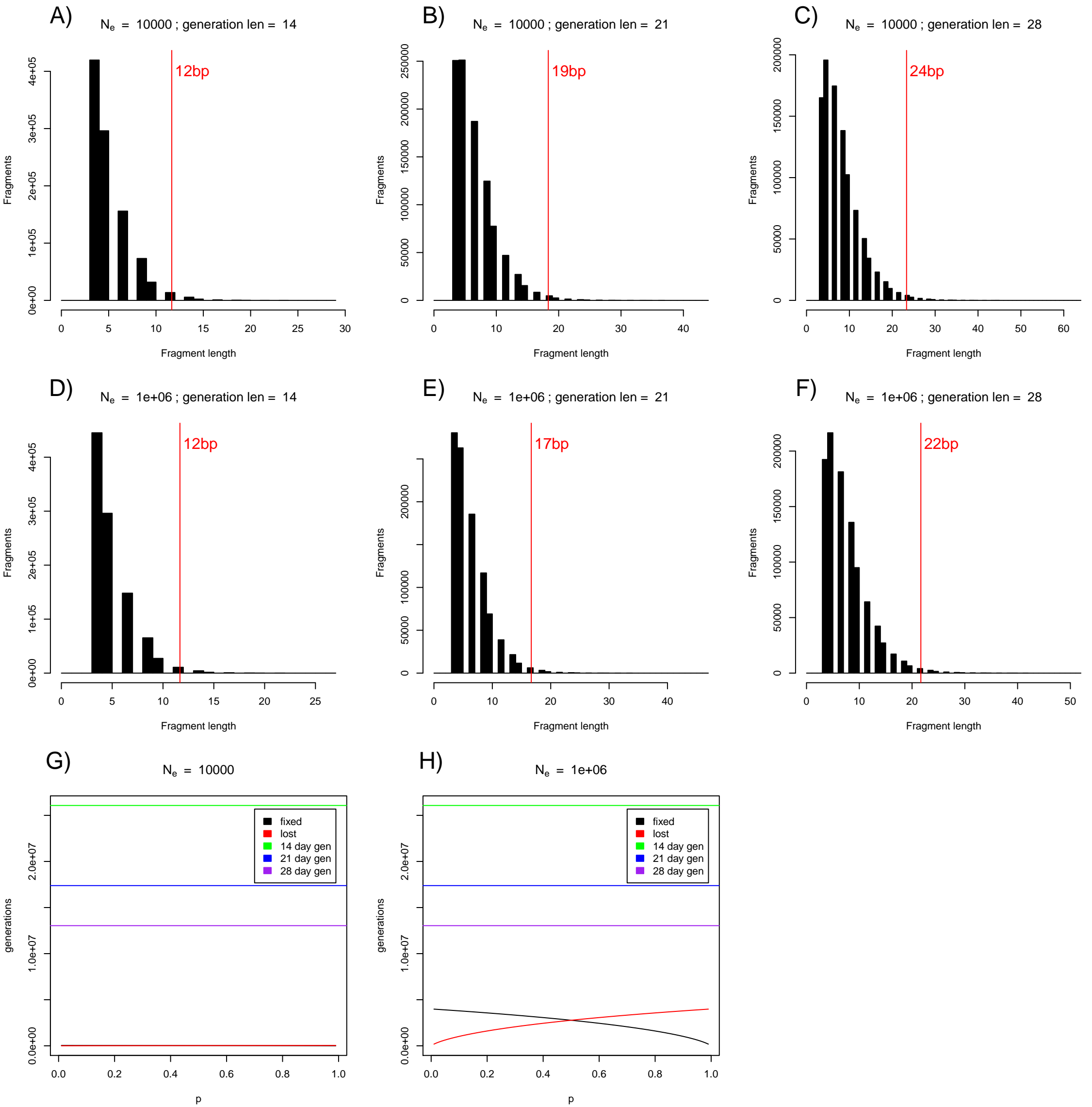

Supplement: S9 Fig — Panels A) through F) show the expected lengths of ancestral haplotype fragments that would still be segregating in the recipient species. The red line and text indicate the 99th quantile of the distribution of fragment sizes. Distributions were calculated assuming different values for the effective population size and generation length. Panels G) and H) show the expected number of generations that an allele at a given frequency p would take to either be fixed (black line) or lost (red line) from the population. Horizontal lines denote the number of populations since the two species diverged assuming generation lengths of 14 days (green line), 21 days (blue line), and 28 days (purple line). A) Ne = 104 and generation length = 14 days. B) Ne = 104 and generation length = 21 days. C) Ne = 104 and generation length = 28 days. D) Ne = 106 and generation length = 14 days. E) Ne = 106 and generation length = 21 days. F) Ne = 106 and generation length = 28 days. G) Ne = 104. H) Ne = 106. (PDF) [file pgen.1006971.s009.pdf]

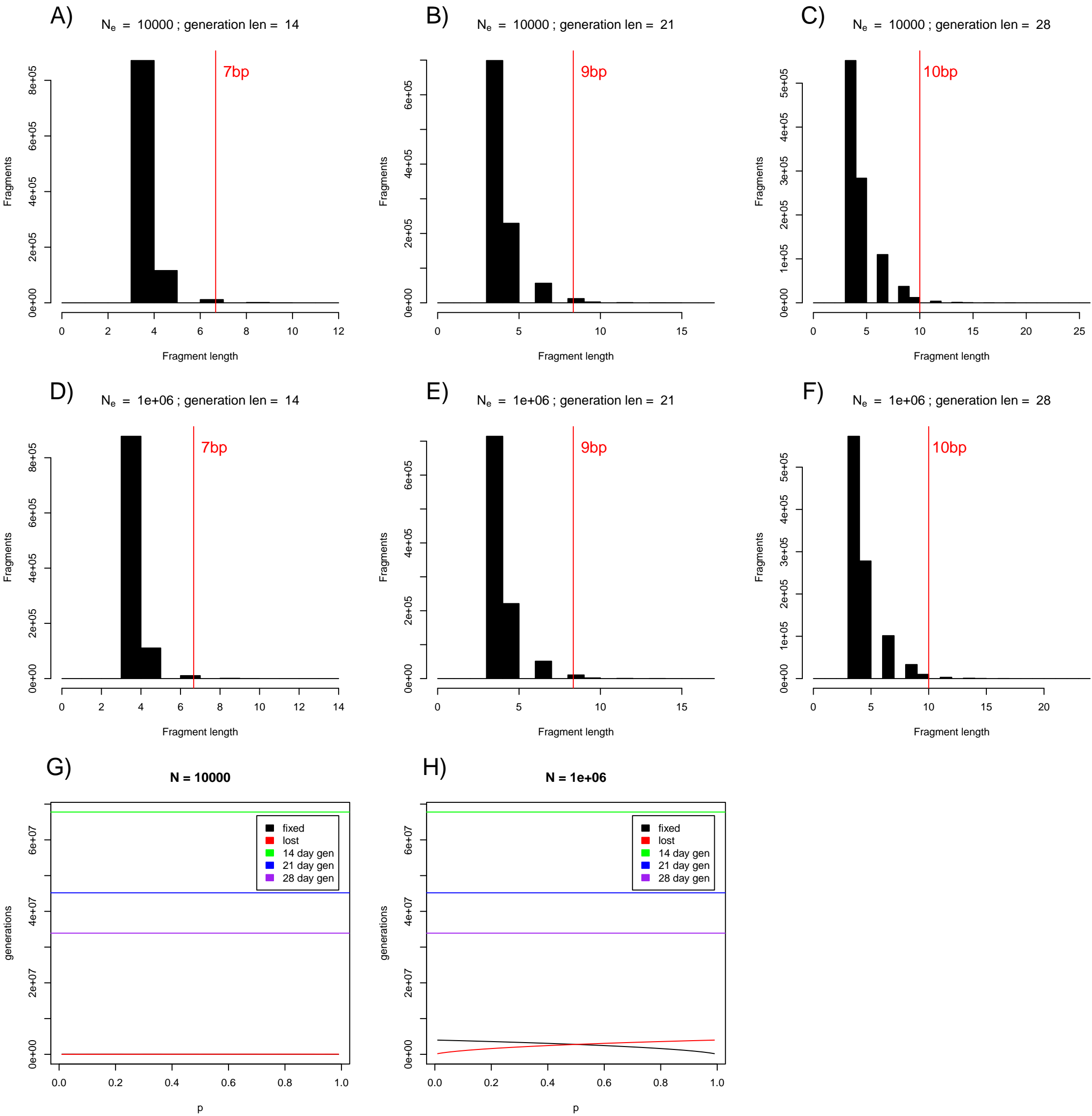

Supplement: S10 Fig — Panels A) through F) show the expected lengths of ancestral haplotype fragments that would still be segregating in the recipient species. The red line and text indicate the 99th quantile of the distribution of fragment sizes. Distributions were calculated assuming different values for the effective population size and generation length. Panels G) and H) show the expected number of generations that an allele at a given frequency p would take to either be fixed (black line) or lost (red line) from the population. Horizontal lines denote the number of populations since the two species diverged assuming generation lengths of 14 days (green line), 21 days (blue line), and 28 days (purple line). A) Ne = 104 and generation length = 14 days. B) Ne = 104 and generation length = 21 days. C) Ne = 104 and generation length = 28 days. D) Ne = 106 and generation length = 14 days. E) Ne = 106 and generation length = 21 days. F) Ne = 106 and generation length = 28 days. G) Ne = 104. H) Ne = 106. (PDF) [file pgen.1006971.s010.pdf]

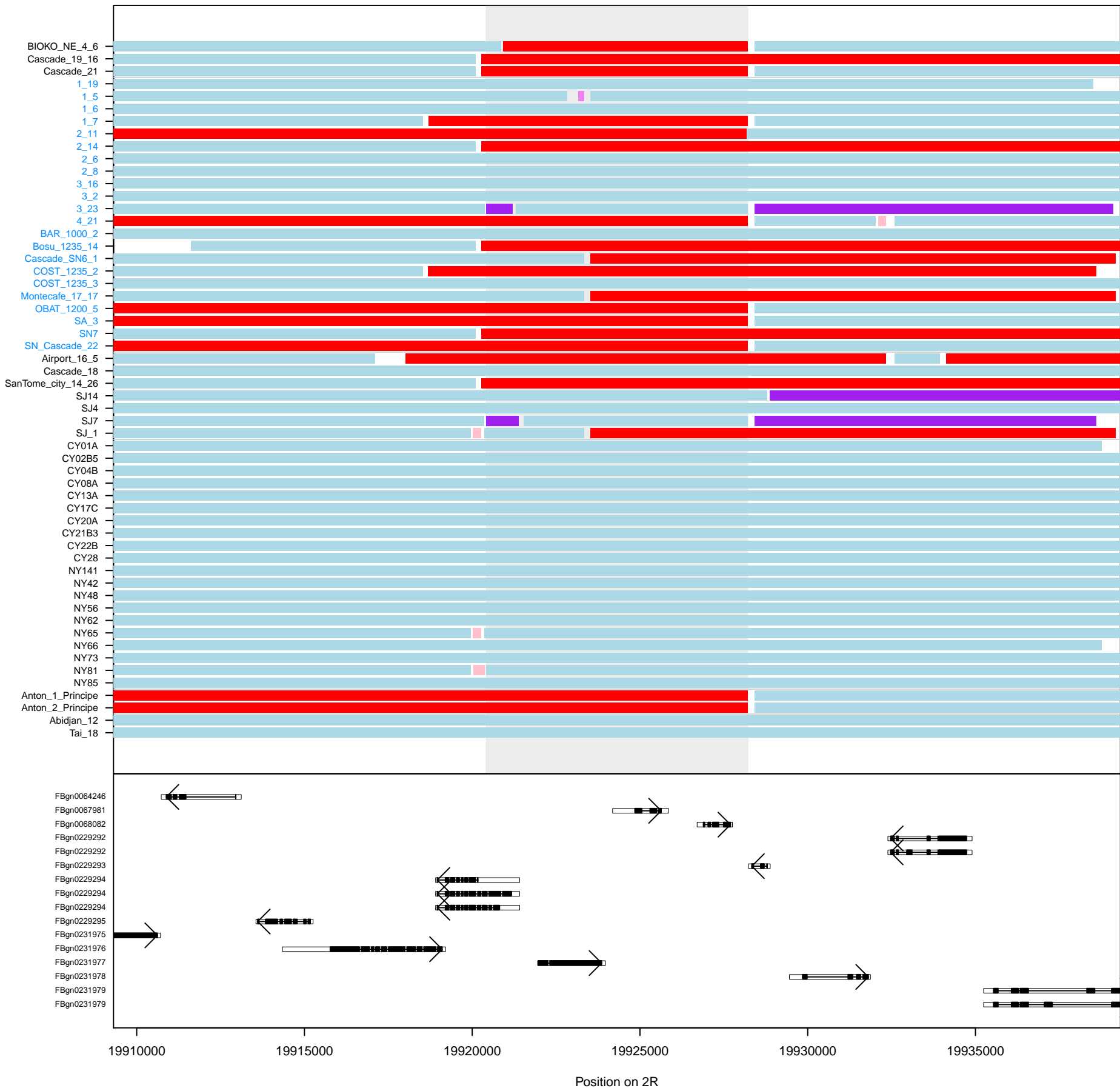

Supplement: S11 Fig — Tracts for all 56 D. yakuba lines. Red bars indicate homozygous D. santomea tracts, purple bars are heterozygous tracts, light bars are homozygous D. yakuba tracts, and light pink tracts indicate homozygous donor tracts that were not considered as introgression tracts because they were either less than 500bp, had less than SNPs with the donor allele, or contained more than 30% repetitive sequence. The region of interest is highlighted by a grey rectangle, and lines from the hybrid zone have blue names. The bottom of the plot contains rectangles indicating annotated genes with an arrow indicating the direction of transcription and solid black rectangles denoting coding sequence. (PDF) [file pgen.1006971.s011.pdf]

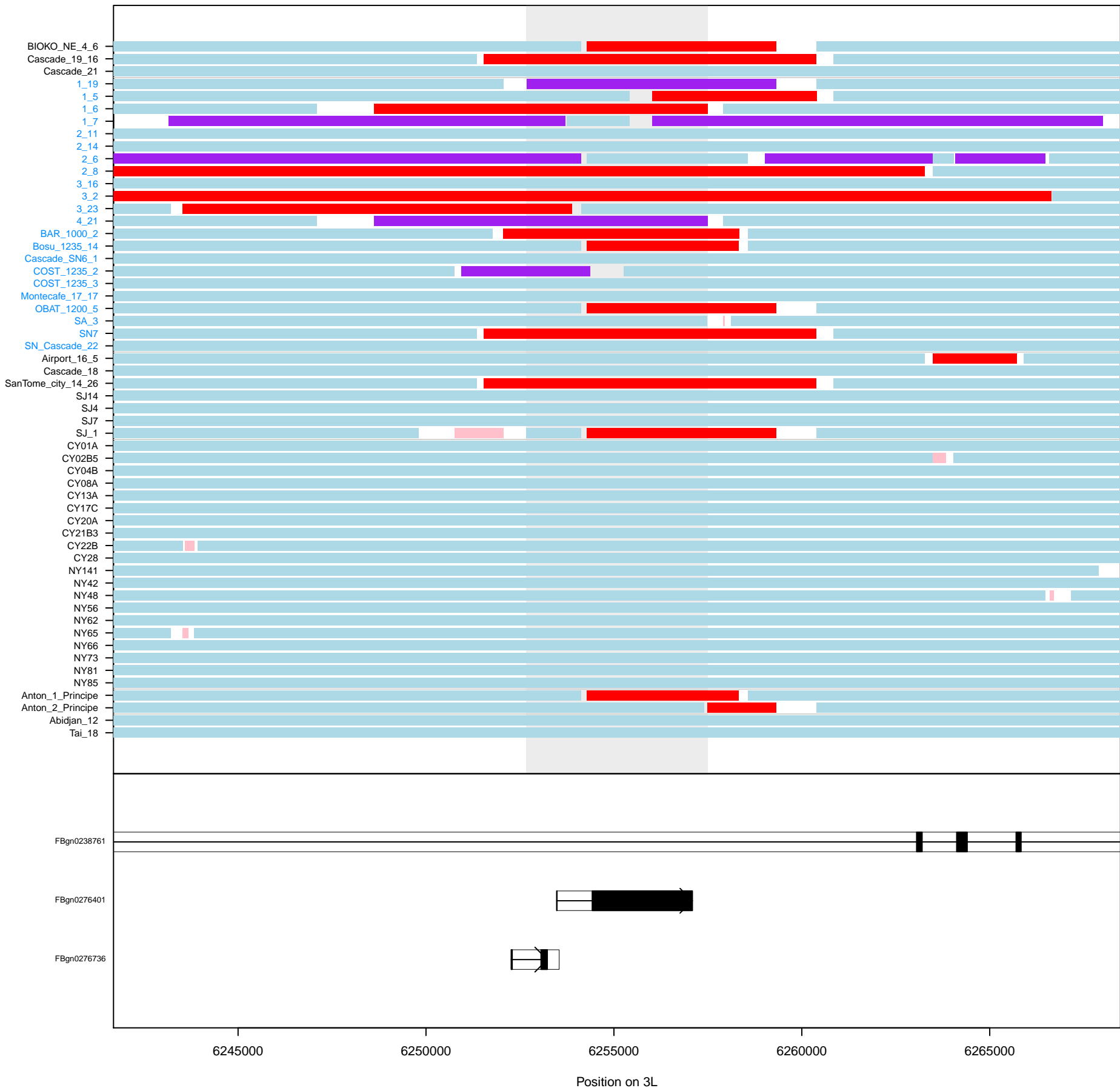

Supplement: S12 Fig — Tracts for all 56 D. yakuba lines. Red bars indicate homozygous D. santomea tracts, purple bars are heterozygous tracts, light bars are homozygous D. yakuba tracts, and light pink tracts indicate homozygous donor tracts that were not considered as introgression tracts because they were either less than 500bp, had less than SNPs with the donor allele, or contained more than 30% repetitive sequence. The region of interest is highlighted by a grey rectangle, and lines from the hybrid zone have blue names. The bottom of the plot contains rectangles indicating annotated genes with an arrow indicating the direction of transcription and solid black rectangles denoting coding sequence. (PDF) [file pgen.1006971.s012.pdf]

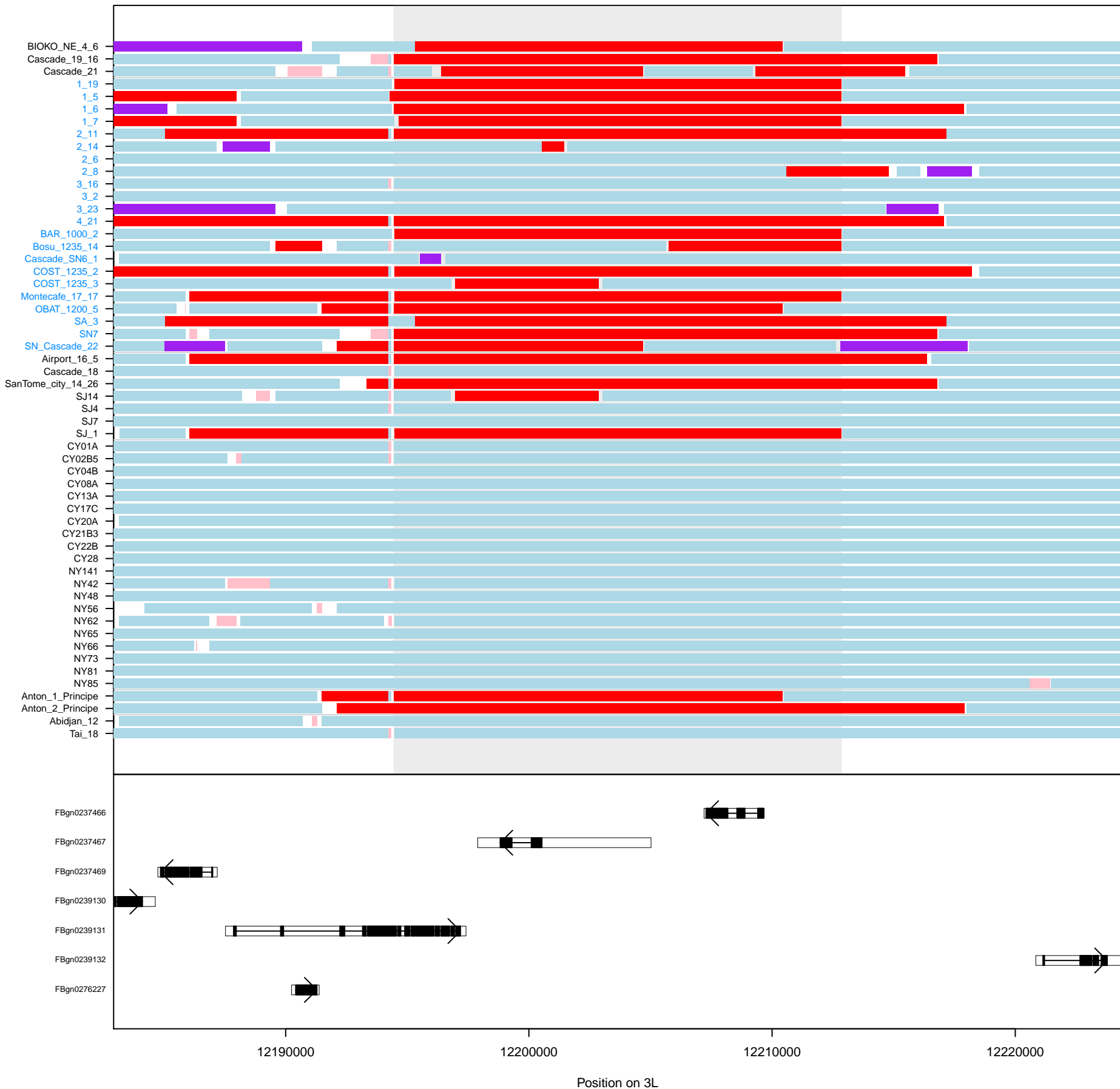

Supplement: S13 Fig — Tracts for all 56 D. yakuba lines. Red bars indicate homozygous D. santomea tracts, purple bars are heterozygous tracts, light bars are homozygous D. yakuba tracts, and light pink tracts indicate homozygous donor tracts that were not considered as introgression tracts because they were either less than 500bp, had less than SNPs with the donor allele, or contained more than 30% repetitive sequence. The region of interest is highlighted by a grey rectangle, and lines from the hybrid zone have blue names. The bottom of the plot contains rectangles indicating annotated genes with an arrow indicating the direction of transcription and solid black rectangles denoting coding sequence. (PDF) [file pgen.1006971.s013.pdf]

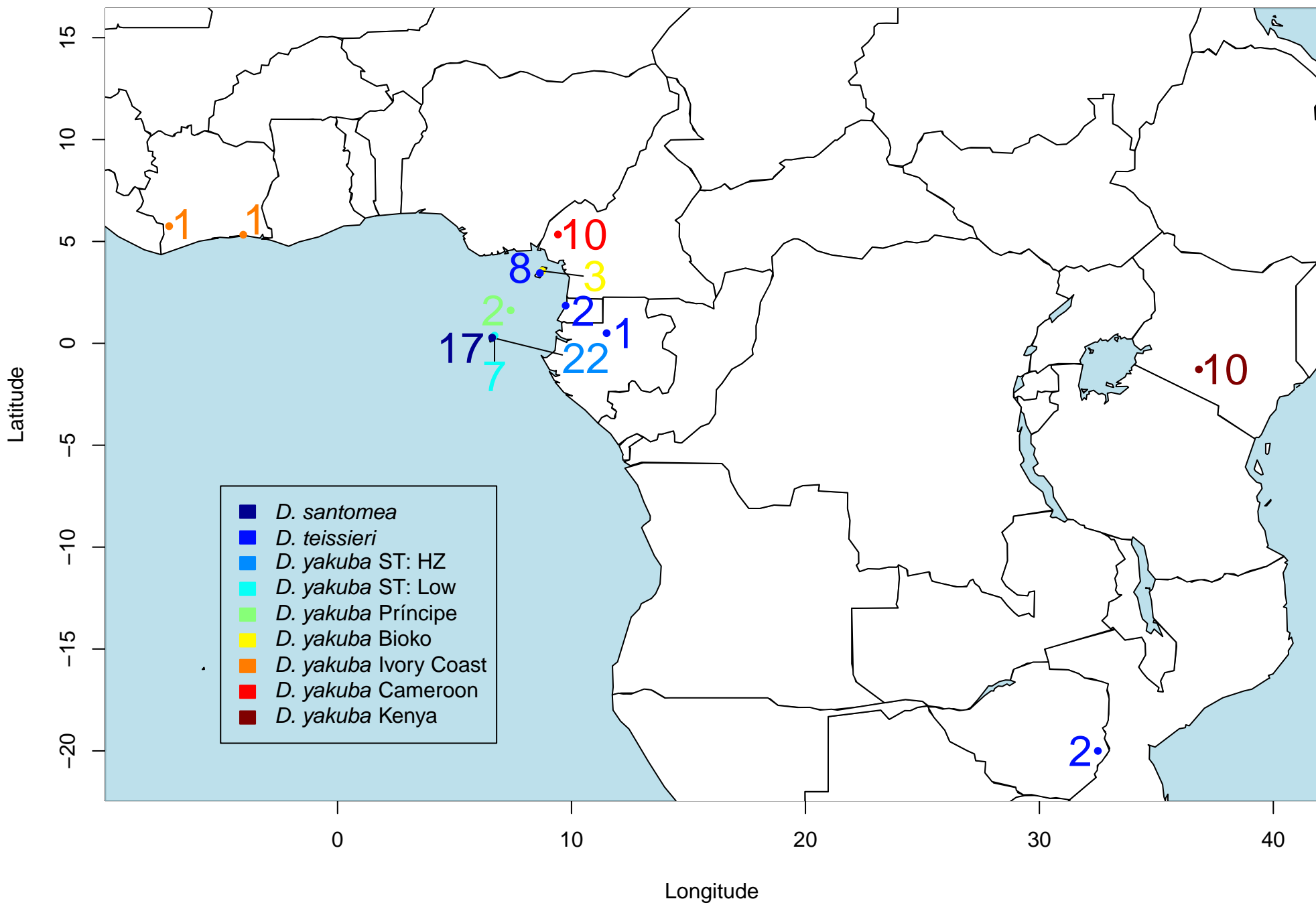

Supplement: S14 Fig — Map indicating the number of fly lines used in this study that were collected from each geographic location. ‘ST: HZ’ is the hybrid zone on São Tomé, and ‘ST: Low’ are the lowland areas on the island of São Tomé. (PDF) [file pgen.1006971.s014.pdf]

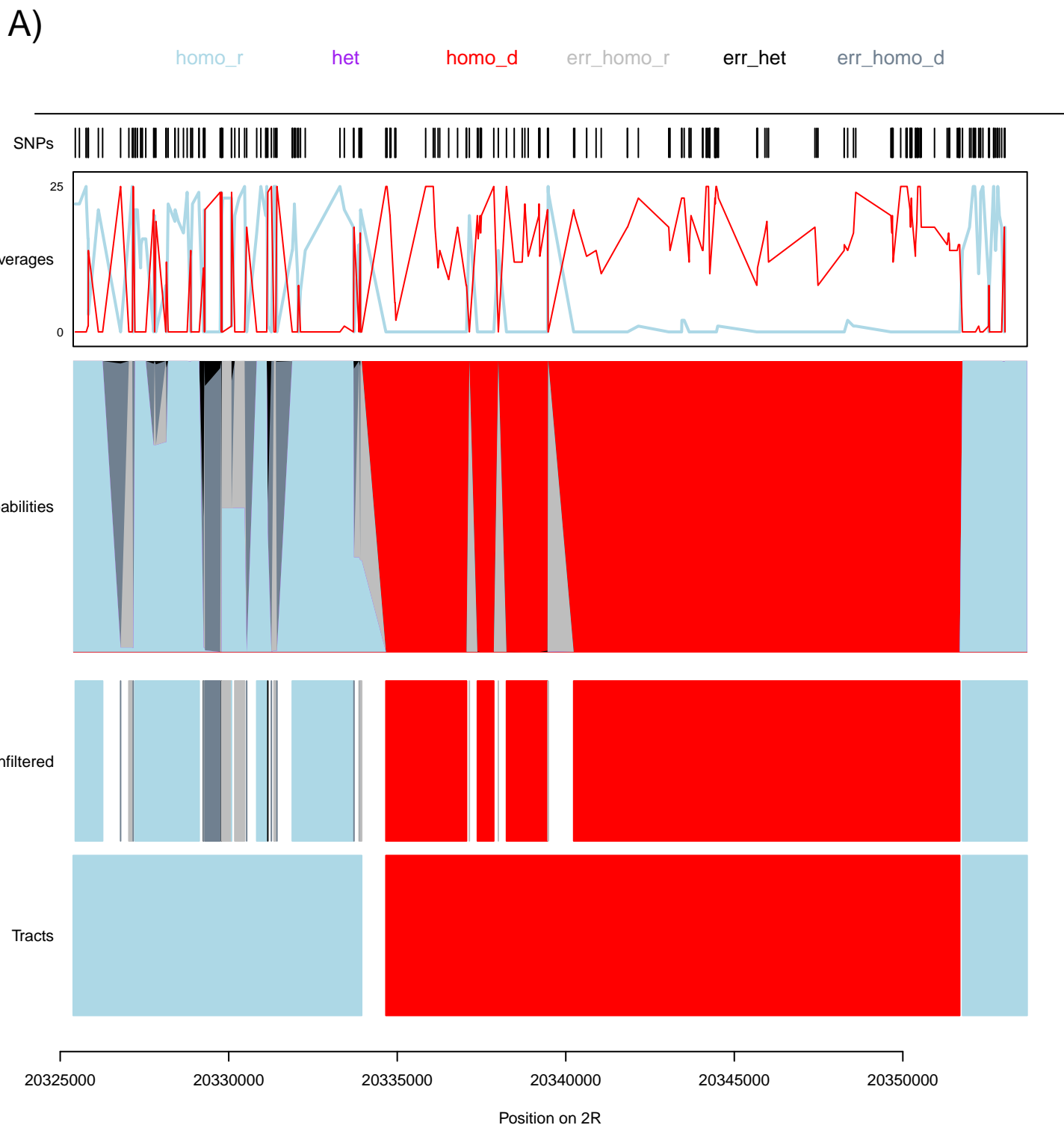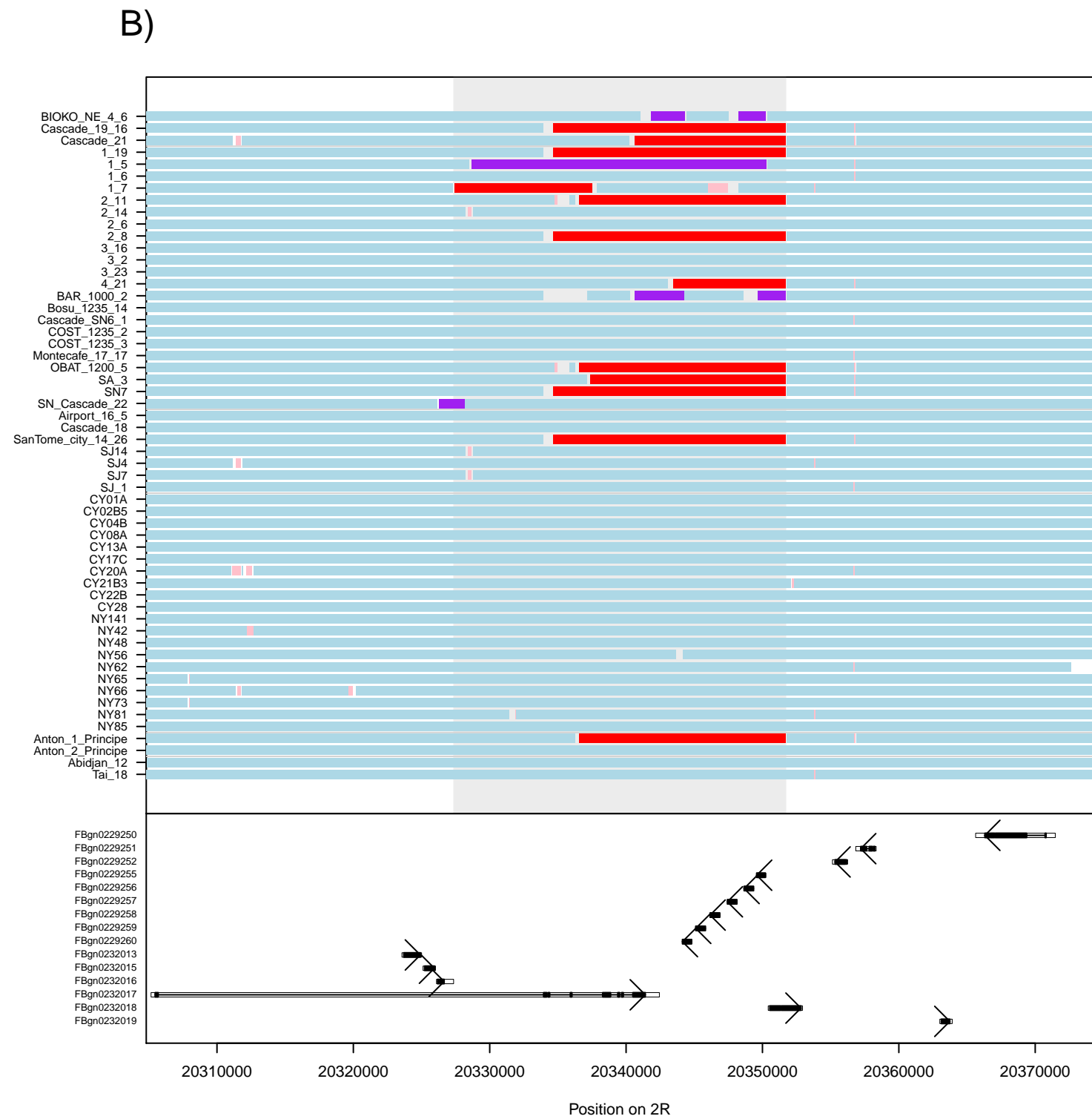

Supplement: S15 Fig — Example introgressions from D. santomea into D. yakuba for a region on chromosome arm 2R. A) Introgression from D. santomea into the D. yakuba line SanTome_city_14_26. ‘SNPs’ represent the markers for this line. ‘Coverages’ show the number of reads with either the donor (D. santomea, red) or recipient allele (D. yakuba, light blue) at each site. Coverages greater than 25x were downscaled to integer values between 0 and 25x. ‘Probabilities’ are the probabilities returned by Int-HMM for all six states at each site (light blue: homozygous recipient, purple: heterozygous, red: homozygous donor, light grey: homozygous recipient error state, black heterozygous error state, and dark grey homozygous donor error state). ‘Unfiltered’ represent the raw tracks obtained by grouping contiguous blocks of SNPs with the same most probable state. ‘Tracts’ are the filtered tracts. B) The same region from A) but showing the filtered tracts for all 56 D. yakuba lines. Light pink tracts indicate homozygous donor tracts that were not considered as introgression tracts because they were either less than 500bp, had less than SNPs with the donor allele, or contained more than 30% repetitive sequence. The bottom of the plot contains rectangles indicating annotated genes with an arrow indicating the direction of transcription and solid black rectangles denoting coding sequence. (PDF) [file pgen.1006971.s015.pdf]
