# Supplementary material for: Fine scale mapping of genomic introgressions within the Drosophila yakuba clade
Source: PLoS Genet. 2017 Sep 5;13(9):e1006971. doi: 10.1371/journal.pgen.1006971 (PMC5600410; doi:10.1371/journal.pgen.1006971)
Supplement: S1 Table — Lines used in the study, their geographic origin, and the length and paired status (se = single end, pe = paired end) of Illumina sequencing reads. Average coverage is the average number of reads mapped overlapping a given site in the genome. The markers columns denote the number of markers used in the HMM when identifying a given direction of introgression. (DOCX) [file pgen.1006971.s016.docx]

**S1 Table. Fly lines used in this study.** Lines used in the study, their geographic origin, and the length and paired status (se = single end, pe = paired end) of Illumina sequencing reads. Average coverage is the average number of reads mapped overlapping a given site in the genome. The markers columns denote the number of markers used in the HMM when identifying a given direction of introgression.

| Species | Population | Line | Pair type | Read length | Average coverage | *yak*-into-*san* markers | *san*-into-*yak* markers | *yak*-into-*tei* markers | *tei*-into-*yak* markers |
| --- | --- | --- | --- | --- | --- | --- | --- | --- | --- |
| *D. santomea* | São Tomé | BS14 | pe | 125 | 98.98 | 923,227 | NA | NA | NA |
| *D. santomea* | São Tomé | C550_39 | pe | 125 | 65.57 | 922,010 | NA | NA | NA |
| *D. santomea* | São Tomé | C650_14 | pe | 125 | 65.58 | 920,827 | NA | NA | NA |
| *D. santomea* | São Tomé | CAR1600 | pe | 125 | 62.54 | 922,131 | NA | NA | NA |
| *D. santomea* | São Tomé | Qiuja630.39 | se | 100 | 24.16 | 913,747 | NA | NA | NA |
| *D. santomea* | São Tomé | Quija37 | se | 100 | 11.74 | 908,062 | NA | NA | NA |
| *D. santomea* | São Tomé | Rain42 | pe | 125 | 69.67 | 919,231 | NA | NA | NA |
| *D. santomea* | São Tomé | sanC1350.14 | se | 100 | 18.62 | 911,619 | NA | NA | NA |
| *D. santomea* | São Tomé | sanCAR1490.5 | se | 100 | 15.77 | 909,499 | NA | NA | NA |
| *D. santomea* | São Tomé | sanCOST1250.5 | se | 100 | 13.27 | 910,422 | NA | NA | NA |
| *D. santomea* | São Tomé | sanCOST1270.6 | se | 100 | 14.76 | 908,533 | NA | NA | NA |
| *D. santomea* | São Tomé | sanOBAT1200.13 | se | 100 | 14.47 | 909,674 | NA | NA | NA |
| *D. santomea* | São Tomé | sanOBAT1200.5 | se | 100 | 16.82 | 908,547 | NA | NA | NA |
| *D. santomea* | São Tomé | sanRain39 | se | 100 | 15.81 | 910,035 | NA | NA | NA |
| *D. santomea* | São Tomé | sanSTO7 | se | 100 | 15.29 | 907,959 | NA | NA | NA |
| *D. santomea* | São Tomé | sanThena5 | se | 100 | 12.98 | 909,543 | NA | NA | NA |
| *D. santomea* | São Tomé | san_Field3 | pe | 125 | 60.78 | 918,936 | NA | NA | NA |
| *D. teissieri* | Bioko | Balancha_1 | pe | 150 | 30.37 | NA | NA | 2,440,335 | NA |
| *D. teissieri* | Bioko | cascade_2_1 | pe | 150 | 29.2 | NA | NA | 2,435,953 | NA |
| *D. teissieri* | Bioko | cascade_2_2 | pe | 150 | 33.88 | NA | NA | 2,439,716 | NA |
| *D. teissieri* | Bioko | cascade_2_4 | pe | 150 | 26.91 | NA | NA | 2,438,424 | NA |
| *D. teissieri* | Bioko | cascade_4_1 | pe | 150 | 27.07 | NA | NA | 2,435,999 | NA |
| *D. teissieri* | Bioko | cascade_4_2 | pe | 150 | 39.54 | NA | NA | 2,468,955 | NA |
| *D. teissieri* | Bioko | cascade_4_3 | pe | 150 | 23.26 | NA | NA | 2,430,334 | NA |
| *D. teissieri* | Bioko | House_Bioko | pe | 150 | 35.7 | NA | NA | 2,445,383 | NA |
| *D. teissieri* | Equatorial Guinea | Bata2 | se | 100 | 20.7 | NA | NA | 2,275,453 | NA |
| *D. teissieri* | Equatorial Guinea | Bata8 | se | 100 | 18.56 | NA | NA | 2,280,660 | NA |
| *D. teissieri* | Gabon | La_Lope_Gabon | pe | 150 | 36.6 | NA | NA | 2,426,237 | NA |
| *D. teissieri* | Zimbabwe | Selinda | pe | 150 | 27.74 | NA | NA | 2,425,028 | NA |
| *D. teissieri* | Zimbabwe | Zimbabwe | pe | 150 | 32.17 | NA | NA | 2,429,980 | NA |
| *D. yakuba* | Bioko | BIOKO_NE_4_6 | se | 101 | 17.17 | NA | 933,776 | NA | 1,867,399 |
| *D. yakuba* | Bioko | Cascade_19_16 | se | 101 | 16.5 | NA | 943,502 | NA | 1,880,181 |
| *D. yakuba* | Bioko | Cascade_21 | se | 101 | 20.59 | NA | 944,109 | NA | 1,880,538 |
| *D. yakuba* | Cameroon | CY01A | pe | 48-76 | 196.72 | NA | 947,830 | NA | 1,884,951 |
| *D. yakuba* | Cameroon | CY02B5 | pe | 48-76 | 69.98 | NA | 947,600 | NA | 1,884,804 |
| *D. yakuba* | Cameroon | CY04B | pe | 48-76 | 157.94 | NA | 947,066 | NA | 1,884,131 |
| *D. yakuba* | Cameroon | CY08A | pe | 48-76 | 75.04 | NA | 947,474 | NA | 1,884,652 |
| *D. yakuba* | Cameroon | CY13A | pe | 48-76 | 72.72 | NA | 946,305 | NA | 1,883,404 |
| *D. yakuba* | Cameroon | CY17C | pe | 48-76 | 193.88 | NA | 947,132 | NA | 1,883,074 |
| *D. yakuba* | Cameroon | CY20A | pe | 76 | 183.65 | NA | 947,522 | NA | 1,884,488 |
| *D. yakuba* | Cameroon | CY21B3 | pe | 48-76 | 173.17 | NA | 948,290 | NA | 1,885,320 |
| *D. yakuba* | Cameroon | CY22B | pe | 54-76 | 69.84 | NA | 945,462 | NA | 1,882,133 |
| *D. yakuba* | Cameroon | CY28 | pe | 54-76 | 110.16 | NA | 946,716 | NA | 1,883,689 |
| *D. yakuba* | São Tomé - hybrid zone | 1_19 | se | 101 | 18.51 | NA | 948,992 | NA | 1,886,150 |
| *D. yakuba* | São Tomé - hybrid zone | 1_5 | se | 101 | 19.27 | NA | 946,264 | NA | 1,883,489 |
| *D. yakuba* | São Tomé - hybrid zone | 1_6 | se | 101 | 20.16 | NA | 946,531 | NA | 1,883,322 |
| *D. yakuba* | São Tomé - hybrid zone | 1_7 | se | 101 | 22.01 | NA | 945,489 | NA | 1,881,957 |
| *D. yakuba* | São Tomé - hybrid zone | 2_11 | se | 101 | 19.51 | NA | 947,314 | NA | 1,884,343 |
| *D. yakuba* | São Tomé - hybrid zone | 2_14 | se | 101 | 19.15 | NA | 943,849 | NA | 1,880,042 |
| *D. yakuba* | São Tomé - hybrid zone | 2_6 | se | 101 | 23.43 | NA | 947,868 | NA | 1,885,046 |
| *D. yakuba* | São Tomé - hybrid zone | 2_8 | se | 101 | 20.38 | NA | 945,740 | NA | 1,882,125 |
| *D. yakuba* | São Tomé - hybrid zone | 3_16 | se | 101 | 19.82 | NA | 950,445 | NA | 1,886,111 |
| *D. yakuba* | São Tomé - hybrid zone | 3_2 | se | 101 | 21.89 | NA | 946,422 | NA | 1,883,168 |
| *D. yakuba* | São Tomé - hybrid zone | 3_23 | se | 101 | 22.11 | NA | 947,241 | NA | 1,883,748 |
| *D. yakuba* | São Tomé - hybrid zone | 4_21 | se | 101 | 22.44 | NA | 945,747 | NA | 1,882,275 |
| *D. yakuba* | São Tomé - hybrid zone | BAR_1000_2 | se | 101 | 21.23 | NA | 946,415 | NA | 1,883,076 |
| *D. yakuba* | São Tomé - hybrid zone | Bosu_1235_14 | se | 101 | 17.22 | NA | 946,208 | NA | 1,882,526 |
| *D. yakuba* | São Tomé - hybrid zone | Cascade_SN6_1 | se | 101 | 18.85 | NA | 946,885 | NA | 1,883,622 |
| *D. yakuba* | São Tomé - hybrid zone | COST_1235_2 | se | 101 | 17.69 | NA | 943,865 | NA | 1,880,498 |
| *D. yakuba* | São Tomé - hybrid zone | COST_1235_3 | se | 101 | 15.42 | NA | 945,360 | NA | 1,881,758 |
| *D. yakuba* | São Tomé - hybrid zone | Montecafe_17_17 | se | 101 | 19.97 | NA | 946,512 | NA | 1,882,615 |
| *D. yakuba* | São Tomé - hybrid zone | OBAT_1200_5 | se | 101 | 22.7 | NA | 944,830 | NA | 1,881,489 |
| *D. yakuba* | São Tomé - hybrid zone | SA_3 | se | 101 | 18.64 | NA | 945,733 | NA | 1,882,474 |
| *D. yakuba* | São Tomé - hybrid zone | SN7 | se | 101 | 23.66 | NA | 943,004 | NA | 1,879,706 |
| *D. yakuba* | São Tomé - hybrid zone | SN_Cascade_22 | se | 101 | 21.78 | NA | 946,489 | NA | 1,883,209 |
| *D. yakuba* | Kenya | NY141 | pe | 54-76 | 143.54 | NA | 945,542 | NA | 1,882,198 |
| *D. yakuba* | Kenya | NY42 | pe | 54-76 | 118.02 | NA | 946,394 | NA | 1,883,219 |
| *D. yakuba* | Kenya | NY48 | pe | 54-76 | 84.99 | NA | 944,685 | NA | 1,881,592 |
| *D. yakuba* | Kenya | NY56 | pe | 54-76 | 88.65 | NA | 944,598 | NA | 1,881,734 |
| *D. yakuba* | Kenya | NY62 | pe | 54-76 | 94.51 | NA | 947,230 | NA | 1,884,258 |
| *D. yakuba* | Kenya | NY65 | pe | 54-76 | 91.46 | NA | 945,551 | NA | 1,882,950 |
| *D. yakuba* | Kenya | NY66 | pe | 54-76 | 148.65 | NA | 945,667 | NA | 1,882,021 |
| *D. yakuba* | Kenya | NY73 | pe | 54-76 | 92.08 | NA | 945,136 | NA | 1,881,926 |
| *D. yakuba* | Kenya | NY81 | pe | 54-76 | 148.42 | NA | 945,534 | NA | 1,882,030 |
| *D. yakuba* | Kenya | NY85 | pe | 54-76 | 99.03 | NA | 947,888 | NA | 1,884,845 |
| *D. yakuba* | São Tomé - lowlands | Airport_16_5 | se | 101 | 20.11 | NA | 947,456 | NA | 1,884,225 |
| *D. yakuba* | São Tomé - lowlands | Cascade_18 | se | 101 | 23.96 | NA | 950,239 | NA | 1,886,650 |
| *D. yakuba* | São Tomé - lowlands | SanTome_city_14_26 | se | 101 | 22.75 | NA | 944,997 | NA | 1,881,836 |
| *D. yakuba* | São Tomé - lowlands | SJ14 | se | 101 | 15.77 | NA | 941,359 | NA | 1,877,179 |
| *D. yakuba* | São Tomé - lowlands | SJ4 | se | 101 | 25.82 | NA | 951,384 | NA | 1,888,413 |
| *D. yakuba* | São Tomé - lowlands | SJ7 | se | 101 | 19.51 | NA | 944,909 | NA | 1,881,298 |
| *D. yakuba* | São Tomé - lowlands | SJ_1 | se | 101 | 21.35 | NA | 946,861 | NA | 1,883,685 |
| *D. yakuba* | Príncipe | Anton_1_Principe | se | 101 | 19.54 | NA | 944,145 | NA | 1,880,516 |
| *D. yakuba* | Príncipe | Anton_2_Principe | se | 101 | 21.38 | NA | 942,331 | NA | 1,878,698 |
| *D. yakuba* | Ivory Coast | Abidjan_12 | se | 101 | 23.79 | NA | 947,025 | NA | 1,884,229 |
| *D. yakuba* | Ivory Coast | Tai_18 | se | 101 | 22.17 | NA | 951,190 | NA | 1,887,883 |
