# Supplementary material for: Fine scale mapping of genomic introgressions within the Drosophila yakuba clade
Source: PLoS Genet. 2017 Sep 5;13(9):e1006971. doi: 10.1371/journal.pgen.1006971 (PMC5600410; doi:10.1371/journal.pgen.1006971)
Supplement: S2 Table — Counts denote the number of tracts, percentages refer to amount of genomic sequence covered by those tracts, and cum lengths are the combined tract lengths. (DOCX) [file pgen.1006971.s017.docx]

**S2 Table. False positive tracts from identified by Int-HMM from the simulated data.** Counts denote the number of tracts, percentages refer to amount of genomic sequence covered by those tracts, and cum lengths are the combined tract lengths.

| direction | homozygous count | homozygous percentage | homozygous cum length | heterozygous count | heterozygous percentage | heterozygous cumulative length |
| --- | --- | --- | --- | --- | --- | --- |
| *yak*-into-*san* | 2 | 0.177 | 2087bp | 0 | 0 | 0bp |
| *san*-into-*yak* | 3 | 0.253 | 5652bp | 0 | 0 | 0bp |
| *yak*-into-*tei* | 1 | 0.0702 | 800bp | 0 | 0 | 0bp |
| *tei*-into-*yak* | 0 | 0 | 0bp | 0 | 0 | 0bp |
