# Supplementary material for: Fine scale mapping of genomic introgressions within the Drosophila yakuba clade
Source: PLoS Genet. 2017 Sep 5;13(9):e1006971. doi: 10.1371/journal.pgen.1006971 (PMC5600410; doi:10.1371/journal.pgen.1006971)
Supplement: S3 Table — Percentage of the genome that was introgressed for each line as determined by the cumulative length of introgression tracts identified by the HMM. (DOCX) [file pgen.1006971.s018.docx]

**S3 Table. Percentage of genome that was introgressed.** Percentage of the genome that was introgressed for each line as determined by the cumulative length of introgression tracts identified by the HMM.

| Species | Population | Line | *yak*-into-*san* | *san*-into-*yak* | *yak*-into-*tei* | *tei*-into-*yak* |
| --- | --- | --- | --- | --- | --- | --- |
| *D. santomea* | São Tomé | Qiuja630.39 | 0.1006 | NA | NA | NA |
| *D. santomea* | São Tomé | Quija37 | 0.4365 | NA | NA | NA |
| *D. santomea* | São Tomé | sanC1350.14 | 0.1221 | NA | NA | NA |
| *D. santomea* | São Tomé | sanCAR1490.5 | 0.5787 | NA | NA | NA |
| *D. santomea* | São Tomé | sanCOST1250.5 | 0.1037 | NA | NA | NA |
| *D. santomea* | São Tomé | sanCOST1270.6 | 0.1698 | NA | NA | NA |
| *D. santomea* | São Tomé | sanOBAT1200.13 | 0.3148 | NA | NA | NA |
| *D. santomea* | São Tomé | sanOBAT1200.5 | 0.1028 | NA | NA | NA |
| *D. santomea* | São Tomé | sanRain39 | 0.1501 | NA | NA | NA |
| *D. santomea* | São Tomé | sanSTO7 | 0.5866 | NA | NA | NA |
| *D. santomea* | São Tomé | sanThena5 | 0.34 | NA | NA | NA |
| *D. santomea* | São Tomé | Rain42 | 0.5293 | NA | NA | NA |
| *D. santomea* | São Tomé | BS14 | 0.4534 | NA | NA | NA |
| *D. santomea* | São Tomé | C650_14 | 0.1293 | NA | NA | NA |
| *D. santomea* | São Tomé | C550_39 | 0.5837 | NA | NA | NA |
| *D. santomea* | São Tomé | san_Field3 | 1.0401 | NA | NA | NA |
| *D. santomea* | São Tomé | CAR1600 | 0.212 | NA | NA | NA |
| *D. teissieri* | Bioko | Balancha_1 | NA | NA | 0.0053 | NA |
| *D. teissieri* | Bioko | cascade_4_3 | NA | NA | 0.0112 | NA |
| *D. teissieri* | Bioko | House_Bioko | NA | NA | 0.0123 | NA |
| *D. teissieri* | Bioko | cascade_4_2 | NA | NA | 0.0107 | NA |
| *D. teissieri* | Bioko | cascade_4_1 | NA | NA | 0.0053 | NA |
| *D. teissieri* | Bioko | cascade_2_4 | NA | NA | 0.0093 | NA |
| *D. teissieri* | Bioko | cascade_2_2 | NA | NA | 0.0052 | NA |
| *D. teissieri* | Bioko | cascade_2_1 | NA | NA | 0 | NA |
| *D. teissieri* | Equatorial Guinea | Bata8 | NA | NA | 0.0008 | NA |
| *D. teissieri* | Equatorial Guinea | Bata2 | NA | NA | 0.004 | NA |
| *D. teissieri* | Gabon | La_Lope_Gabon | NA | NA | 0.0065 | NA |
| *D. teissieri* | Zimbabwe | Zimbabwe | NA | NA | 0.006 | NA |
| *D. teissieri* | Zimbabwe | Selinda | NA | NA | 0.0129 | NA |
| *D. yakuba* | Bioko | BIOKO_NE_4_6 | NA | 0.2751 | NA | 0.0088 |
| *D. yakuba* | Bioko | Cascade_19_16 | NA | 0.3349 | NA | 0.0144 |
| *D. yakuba* | Bioko | Cascade_21 | NA | 0.321 | NA | 0.0011 |
| *D. yakuba* | Cameroon | CY28 | NA | 0.1097 | NA | 0.0089 |
| *D. yakuba* | Cameroon | CY22B | NA | 0.0688 | NA | 0.0007 |
| *D. yakuba* | Cameroon | CY21B3 | NA | 0.0146 | NA | 0.0054 |
| *D. yakuba* | Cameroon | CY20A | NA | 0.0919 | NA | 0.0215 |
| *D. yakuba* | Cameroon | CY17C | NA | 0.0168 | NA | 0.001 |
| *D. yakuba* | Cameroon | CY13A | NA | 0.0922 | NA | 0.0073 |
| *D. yakuba* | Cameroon | CY08A | NA | 0.031 | NA | 0.0137 |
| *D. yakuba* | Cameroon | CY04B | NA | 0.0492 | NA | 0.0144 |
| *D. yakuba* | Cameroon | CY01A | NA | 0.0483 | NA | 0.019 |
| *D. yakuba* | Cameroon | CY02B5 | NA | 0.0342 | NA | 0.0084 |
| *D. yakuba* | São Tomé - hybrid zone | Cascade_SN6_1 | NA | 0.3078 | NA | 0.0032 |
| *D. yakuba* | São Tomé - hybrid zone | SN7 | NA | 0.3645 | NA | 0.0144 |
| *D. yakuba* | São Tomé - hybrid zone | SN_Cascade_22 | NA | 0.3039 | NA | 0.0175 |
| *D. yakuba* | São Tomé - hybrid zone | 1_19 | NA | 0.3896 | NA | 0.0065 |
| *D. yakuba* | São Tomé - hybrid zone | 1_5 | NA | 0.3047 | NA | 0.0138 |
| *D. yakuba* | São Tomé - hybrid zone | 1_6 | NA | 0.339 | NA | 0.0129 |
| *D. yakuba* | São Tomé - hybrid zone | 1_7 | NA | 0.3717 | NA | 0.0124 |
| *D. yakuba* | São Tomé - hybrid zone | 2_11 | NA | 0.2351 | NA | 0.0107 |
| *D. yakuba* | São Tomé - hybrid zone | 2_14 | NA | 0.2357 | NA | 0.0007 |
| *D. yakuba* | São Tomé - hybrid zone | 2_6 | NA | 0.3771 | NA | 0.006 |
| *D. yakuba* | São Tomé - hybrid zone | 2_8 | NA | 0.357 | NA | 0.0244 |
| *D. yakuba* | São Tomé - hybrid zone | 3_16 | NA | 0.012 | NA | 0.0176 |
| *D. yakuba* | São Tomé - hybrid zone | 3_2 | NA | 0.4387 | NA | 0.0101 |
| *D. yakuba* | São Tomé - hybrid zone | 3_23 | NA | 0.4047 | NA | 0.0073 |
| *D. yakuba* | São Tomé - hybrid zone | 4_21 | NA | 0.3901 | NA | 0.0153 |
| *D. yakuba* | São Tomé - hybrid zone | COST_1235_2 | NA | 0.304 | NA | 0.0014 |
| *D. yakuba* | São Tomé - hybrid zone | COST_1235_3 | NA | 0.1677 | NA | 0.004 |
| *D. yakuba* | São Tomé - hybrid zone | Montecafe_17_17 | NA | 0.3496 | NA | 0 |
| *D. yakuba* | São Tomé - hybrid zone | BAR_1000_2 | NA | 0.3118 | NA | 0.0072 |
| *D. yakuba* | São Tomé - hybrid zone | Bosu_1235_14 | NA | 0.3575 | NA | 0.0008 |
| *D. yakuba* | São Tomé - hybrid zone | OBAT_1200_5 | NA | 0.3394 | NA | 0.0072 |
| *D. yakuba* | São Tomé - hybrid zone | SA_3 | NA | 0.2419 | NA | 0.0053 |
| *D. yakuba* | Kenya | NY81 | NA | 0.1487 | NA | 0.0084 |
| *D. yakuba* | Kenya | NY85 | NA | 0.0211 | NA | 0.0008 |
| *D. yakuba* | Kenya | NY66 | NA | 0.1322 | NA | 0.0049 |
| *D. yakuba* | Kenya | NY65 | NA | 0.0813 | NA | 0.0036 |
| *D. yakuba* | Kenya | NY62 | NA | 0.0667 | NA | 0.0069 |
| *D. yakuba* | Kenya | NY42 | NA | 0.0821 | NA | 0.0097 |
| *D. yakuba* | Kenya | NY48 | NA | 0.0608 | NA | 0.0092 |
| *D. yakuba* | Kenya | NY73 | NA | 0.0581 | NA | 0.0074 |
| *D. yakuba* | Kenya | NY56 | NA | 0.0739 | NA | 0.001 |
| *D. yakuba* | Kenya | NY141 | NA | 0.0301 | NA | 0.0084 |
| *D. yakuba* | São Tomé - lowlands | Airport_16_5 | NA | 0.2525 | NA | 0.0021 |
| *D. yakuba* | São Tomé - lowlands | Cascade_18 | NA | 0.013 | NA | 0.0114 |
| *D. yakuba* | São Tomé - lowlands | SanTome_city_14_26 | NA | 0.3421 | NA | 0.0133 |
| *D. yakuba* | São Tomé - lowlands | SJ14 | NA | 0.171 | NA | 0.0044 |
| *D. yakuba* | São Tomé - lowlands | SJ4 | NA | 0.0264 | NA | 0.0087 |
| *D. yakuba* | São Tomé - lowlands | SJ7 | NA | 0.2344 | NA | 0.0017 |
| *D. yakuba* | São Tomé - lowlands | SJ_1 | NA | 0.3074 | NA | 0 |
| *D. yakuba* | Príncipe | Anton_1_Principe | NA | 0.3142 | NA | 0.0072 |
| *D. yakuba* | Príncipe | Anton_2_Principe | NA | 1.2012 | NA | 0 |
| *D. yakuba* | Ivory Coast | Tai_18 | NA | 0.0182 | NA | 0.0103 |
| *D. yakuba* | Ivory Coast | Abidjan_12 | NA | 0.1756 | NA | 0.0143 |
