# Supplementary material for: Fine scale mapping of genomic introgressions within the Drosophila yakuba clade
Source: PLoS Genet. 2017 Sep 5;13(9):e1006971. doi: 10.1371/journal.pgen.1006971 (PMC5600410; doi:10.1371/journal.pgen.1006971)
Supplement: S4 Table — (DOCX) [file pgen.1006971.s019.docx]

**S4 Table. Distribution of markers used by Int-HMM is similar across sequence types in all the four introgressions directions.**

| **Direction** | **type** | **markers** | **total** | **Markers per kb** |
| --- | --- | --- | --- | --- |
| *yak*-into-*san* | 10kb inter | 166,112 | 22,780,104 | 7.292 |
| *yak*-into-*san* | 2kb upstream inter | 92,911 | 10,100,186 | 9.1989 |
| *yak*-into-*san* | 3’prime UTR | 31,596 | 4,121,972 | 7.6653 |
| *yak*-into-*san* | 5’prime UTR | 27,077 | 3,346,831 | 8.0903 |
| *yak*-into-*san* | CDS | 145,211 | 22,059,257 | 6.5828 |
| *yak*-into-*san* | exon | 2,375 | 317,599 | 7.478 |
| *yak*-into-*san* | intergenic | 50,263 | 8,754,040 | 5.7417 |
| *yak*-into-*san* | intron | 415,743 | 48,178,319 | 8.6293 |
| *san*-into-*yak* | 10kb inter | 166,866 | 22,780,104 | 7.3251 |
| *san*-into-*yak* | 2kb upstream inter | 98,371 | 10,100,186 | 9.7395 |
| *san*-into-*yak* | 3’prime UTR | 30,881 | 4,121,972 | 7.4918 |
| *san*-into-*yak* | 5’prime UTR | 26,267 | 3,346,831 | 7.8483 |
| *san*-into-*yak* | CDS | 156,748 | 22,059,257 | 7.1058 |
| *san*-into-*yak* | exon | 2,207 | 317,599 | 6.949 |
| *san*-into-*yak* | intergenic | 49,851 | 8,754,040 | 5.6946 |
| *san*-into-*yak* | intron | 421,513 | 48,178,319 | 8.749 |
| *yak*-into-*tei* | 10kb inter | 435,107 | 22,780,104 | 19.1003 |
| *yak*-into-*tei* | 2kb upstream inter | 237,045 | 10,100,186 | 23.4694 |
| *yak*-into-*tei* | 3’prime UTR | 83,077 | 4,121,972 | 20.1547 |
| *yak*-into-*tei* | 5’prime UTR | 74,830 | 3,346,831 | 22.3585 |
| *yak*-into-*tei* | CDS | 475,558 | 22,059,257 | 21.5582 |
| *yak*-into-*tei* | exon | 6,035 | 317,599 | 19.0019 |
| *yak*-into-*tei* | intergenic | 131,295 | 8,754,040 | 14.9982 |
| *yak*-into-*tei* | intron | 1,105,338 | 48,178,319 | 22.9426 |
| *tei*-into-*yak* | 10kb inter | 307,221 | 22,780,104 | 13.4864 |
| *tei*-into-*yak* | 2kb upstream inter | 168,122 | 10,100,186 | 16.6454 |
| *tei*-into-*yak* | 3’prime UTR | 67,187 | 4,121,972 | 16.2997 |
| *tei*-into-*yak* | 5’prime UTR | 61,183 | 3,346,831 | 18.2809 |
| *tei*-into-*yak* | CDS | 401,401 | 22,059,257 | 18.1965 |
| *tei*-into-*yak* | exon | 4,589 | 317,599 | 14.449 |
| *tei*-into-*yak* | intergenic | 91,980 | 8,754,040 | 10.5071 |
| *tei*-into-*yak* | intron | 788,765 | 48,178,319 | 16.3718 |
