# Supplementary material for: Fine scale mapping of genomic introgressions within the Drosophila yakuba clade
Source: PLoS Genet. 2017 Sep 5;13(9):e1006971. doi: 10.1371/journal.pgen.1006971 (PMC5600410; doi:10.1371/journal.pgen.1006971)
Supplement: S5 Table — The genome was partitioned by sequence type with each region being assigned to a single sequence type with the following hierarchy: CDS (coding sequence), exon, 5prime UTR, 3prime UTR, intron, 2kb upstream inter (intergenic sequence 2kb upstream of a gene), 10kb inter (intergenic sequence within 10kb of a gene), and intergenic (intergenic sequence more than 10kb from a gene). ‘Introgressed percentage’ is the percentage of introgressions overlapping a given sequence type for that direction, ‘Genomic percentage’ is the percentage of the genome represented by a given sequence type, and ‘Enrichment’ = (Introgressed percentage) / (Genomic percentage). P-values were calculated with permutation tests as described in the Methods. (DOCX) [file pgen.1006971.s020.docx]

**S5 Table. Sequence types containing introgressions.** The genome was partitioned by sequence type with each region being assigned to a single sequence type with the following hierarchy: CDS (coding sequence), exon, 5prime UTR, 3prime UTR, intron, 2kb upstream inter (intergenic sequence 2kb upstream of a gene), 10kb inter (intergenic sequence within 10kb of a gene), and intergenic (intergenic sequence more than 10kb from a gene). ‘Introgressed percentage’ is the percentage of introgressions overlapping a given sequence type for that direction, ‘Genomic percentage’ is the percentage of the genome represented by a given sequence type, and ‘Enrichment’ = (Introgressed percentage) / (Genomic percentage). P-values were calculated with permutation tests as described in the Methods.

| Direction | Sequence type | Length (kb) | Introgressed percentage | Genomic percentage | Enrichment | P-value |
| --- | --- | --- | --- | --- | --- | --- |
| *yak*-into-*san* | 10kb inter | 553.5 | 23.5 | 19.0 | 1.23 | 0.0020 |
| *yak*-into-*san* | 2kb upstream inter | 130.1 | 5.5 | 8.5 | 0.65 | 0.1980 |
| *yak*-into-*san* | 3prime UTR | 23.4 | 1.0 | 3.5 | 0.29 | 0.0010 |
| *yak*-into-*san* | 5prime UTR | 6.4 | 0.3 | 2.8 | 0.10 | 0.3020 |
| *yak*-into-*san* | CDS | 148.8 | 6.3 | 18.4 | 0.34 | 0.2960 |
| *yak*-into-*san* | exon | 0.2 | 0.0 | 0.3 | 0.03 | 0.2310 |
| *yak*-into-*san* | intergenic | 333.2 | 14.1 | 7.4 | 1.92 | 0.7320 |
| *yak*-into-*san* | intron | 1160.3 | 49.3 | 40.2 | 1.23 | 0.9910 |
|  |  |  |  |  |  |  |
| *san*-into-*yak* | 10kb inter | 611.8 | 18.2 | 19.0 | 0.96 | 0.8110 |
| *san*-into-*yak* | 2kb upstream inter | 166.8 | 5.0 | 8.5 | 0.59 | 0.7940 |
| *san*-into-*yak* | 3prime UTR | 33.1 | 1.0 | 3.5 | 0.28 | 0.0360 |
| *san*-into-*yak* | 5prime UTR | 18.7 | 0.6 | 2.8 | 0.20 | 0.0050 |
| *san*-into-*yak* | CDS | 252.5 | 7.5 | 18.4 | 0.41 | 0.0440 |
| *san*-into-*yak* | exon | 2.1 | 0.1 | 0.3 | 0.23 | 0.0120 |
| *san*-into-*yak* | intergenic | 403.4 | 12.0 | 7.4 | 1.63 | 0.9550 |
| *san*-into-*yak* | intron | 1869.2 | 55.7 | 40.2 | 1.39 | 0.1090 |
|  |  |  |  |  |  |  |
| *yak*-into-*tei* | 10kb inter | 18.0 | 31.0 | 19.0 | 1.63 | 0.5630 |
| *yak*-into-*tei* | 2kb upstream inter | 10.3 | 17.7 | 8.5 | 2.10 | 0.9510 |
| *yak*-into-*tei* | 3prime UTR | 0.3 | 0.5 | 3.5 | 0.15 | 0.1570 |
| *yak*-into-*tei* | 5prime UTR | 0.3 | 0.5 | 2.8 | 0.18 | 0.7430 |
| *yak*-into-*tei* | CDS | 9.7 | 16.6 | 18.4 | 0.90 | 1.0000 |
| *yak*-into-*tei* | exon | 0.0 | 0.0 | 0.3 | 0.00 | 0.0000 |
| *yak*-into-*tei* | intergenic | 4.1 | 7.0 | 7.4 | 0.94 | 0.0470 |
| *yak*-into-*tei* | intron | 15.6 | 26.7 | 40.2 | 0.66 | 0.0000 |
|  |  |  |  |  |  |  |
| *tei*-into-*yak* | 10kb inter | 14.5 | 22.5 | 19.0 | 1.18 | 0.8800 |
| *tei*-into-*yak* | 2kb upstream inter | 8.1 | 12.6 | 8.5 | 1.49 | 0.9940 |
| *tei*-into-*yak* | 3prime UTR | 0.6 | 0.9 | 3.5 | 0.25 | 0.0510 |
| *tei*-into-*yak* | 5prime UTR | 1.0 | 1.6 | 2.8 | 0.56 | 0.1960 |
| *tei*-into-*yak* | CDS | 18.5 | 28.6 | 18.4 | 1.55 | 0.8940 |
| *tei*-into-*yak* | exon | 0.0 | 0.0 | 0.3 | 0.00 | 0.0000 |
| *tei*-into-*yak* | intergenic | 2.3 | 3.6 | 7.4 | 0.49 | 0.6500 |
| *tei*-into-*yak* | intron | 19.5 | 30.3 | 40.2 | 0.75 | 0.0020 |
